# Exposure to high dose of polystyrene nanoplastics causes trophoblast cell apoptosis and induces miscarriage

**DOI:** 10.1186/s12989-024-00574-w

**Published:** 2024-03-07

**Authors:** Shukun Wan, Xiaoqing Wang, Weina Chen, Manli Wang, Jingsong Zhao, Zhongyan Xu, Rong Wang, Chenyang Mi, Zhaodian Zheng, Huidong Zhang

**Affiliations:** 1 Research Center for Environment and Female Reproductive Health, the Eighth Affiliated Hospital, Sun Yat-sen University, 518033 Shenzhen, China; 2 Key Laboratory of Environment and Female Reproductive Health, West China School of Public Health & West China Fourth Hospital, Sichuan University, 610041 Chengdu, China

**Keywords:** Polystyrene nanoplastics, Trophoblast, Apoptosis, Miscarriage

## Abstract

**Background:**

With rapid increase in the global use of various plastics, microplastics (MPs) and nanoplastics (NPs) pollution and their adverse health effects have attracted global attention. MPs have been detected out in human body and both MPs and NPs showed female reproductive toxicological effects in animal models. Miscarriage (abnormal early embryo loss), accounting for 15-25% pregnant women worldwide, greatly harms human reproduction. However, the adverse effects of NPs on miscarriage have never been explored.

**Results:**

In this study, we identified that polystyrene (PS) plastics particles were present in women villous tissues. Their levels were higher in villous tissues of unexplained recurrent miscarriage (RM) patients vs. healthy control (HC) group. Furthermore, mouse assays further confirmed that exposure to polystyrene nanoplastics (PS-NPs, 50 nm in diameter, 50 or 100 mg/kg) indeed induced miscarriage. In mechanism, PS-NPs exposure (50, 100, 150, or 200 µg/mL) increased oxidative stress, decreased mitochondrial membrane potential, and increased apoptosis in human trophoblast cells by activating Bcl-2/Cleaved-caspase-2/Cleaved-caspase-3 signaling through mitochondrial pathway. The alteration in this signaling was consistent in placental tissues of PS-NPs-exposed mouse model and in villous tissues of unexplained RM patients. Supplement with Bcl-2 could efficiently suppress apoptosis in PS-NPs-exposed trophoblast cells and reduce apoptosis and alleviate miscarriage in PS-NPs-exposed pregnant mouse model.

**Conclusions:**

Exposure to PS-NPs activated Bcl-2/Cleaved-caspase-2/Cleaved-caspase-3, leading to excessive apoptosis in human trophoblast cells and in mice placental tissues, further inducing miscarriage.

**Graphical Abstract:**

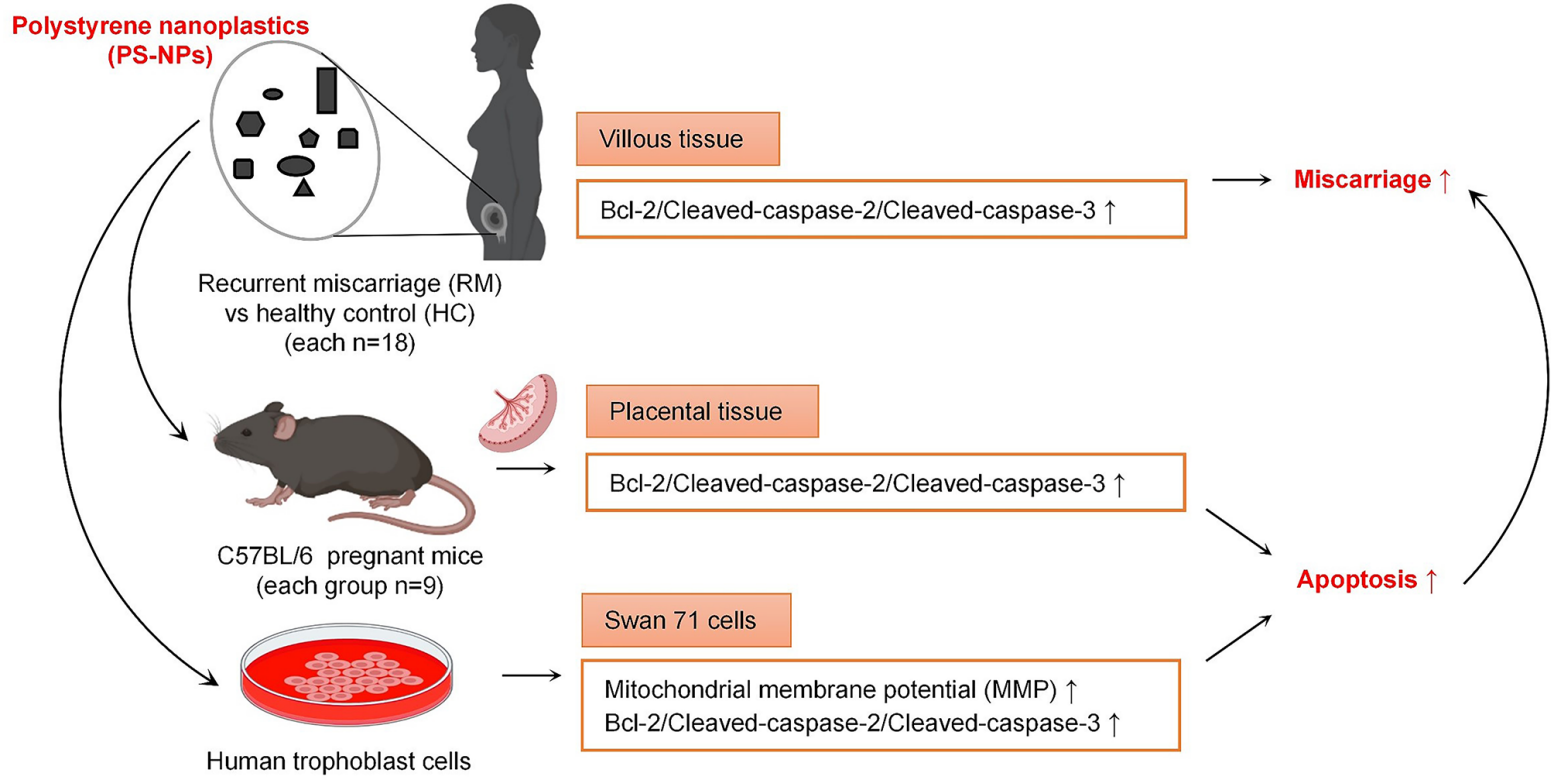

**Supplementary Information:**

The online version contains supplementary material available at 10.1186/s12989-024-00574-w.

## Introduction

### Microplastics and nanoplastics in environment and in human body

In the last century, the global production of various plastics has reached 320 million tons per year, and over 40% is used as single-use packaging, hence producing a huge amount of plastic wastes [1]. However, only approximately 9% plastic wastes could be recycled, and the rest are finally discarded into environments [2]. Atmospheric waves, abrasion, ultraviolet radiation, photo-oxidation, and bacteria behaviors could degrade plastic fragments into micro- and nano-sized particles with different shapes and sizes [3]. In general, plastic particles < 5 mm in diameter are classified as microplastics (MPs) [4] and those within 1-100 nm are considered as nanoplastics (NPs) [5]. The main components of MPs are polystyrene (PS), polyethylene terephthalate (PET), polyethylene (PE), polypropylene (PP), and polyvinyl chloride (PVC) in the environment, among which PS is the predominant type [6, 7]. PS is regarded as one of the most common types of plastic debris in coastal marine environments [8, 9]. For example, among 120 samples collected from the southern Adriatic Sea, 80.6% contained plastic debris, of which 38.7% was polystyrene [10]. PS-MPs or PS-NPs have been widely selected as model and used in various studies.

Recently, increasing studies have shown that MPs were detected out from food (particularly seafood) [11], sea salt [12, 13], drinking water [14], and atmospheric fallout [15]. Due to food-chain transportation, drinks, and inhalation, plastic particles were also detected out from human blood [16], human stools [17], colectomy specimens [18], and lungs [19]. A report has shown that the median intake of MPs (1-5000 μm) is estimated as 4.1 µg/week (or 0.58 µg/day) for an adult by correcting their actual contents in foods [20]. The daily intake of MPs with diameters between 0.5 and 10 μm is estimated as 40.1 µg/kg body weight/day from bottled mineral waters [21]. MPs are accumulated in gastrointestinal tract, liver, kidney, and brain of mammals and then induced oxidative stress and inflammation response, interfered lipid and energy metabolism, and altered blood biomarkers and neurotoxicity [22–24]. Collectively, these studies raise the concerns about the long-term adverse effects of plastics particles on human health.

### Microplastics and nanoplastics produce female reproductive toxicity

It has been shown that MPs cause reproductive disruption in Daphnia, oyster, and Ceriodaphnia dubia [25–27]. PS-MPs exposure induces ovarian inflammation and reduces oocyte quality in mice [28], as well as induces ovarian fibrosis, apoptosis, and pyroptosis of granulosa cells in female rats [29]. MPs (5–10 μm in size) have been identified for the first time in placenta of pregnant women, including the fetal side, the maternal side, and the chorioamniotic membranes [30]. Compared with MPs, NPs might have greater potential toxic effects on reproduction because NPs can more readily enter tissues and organs by crossing biological barriers due to their smaller sizes [31]. It has been reported that PS-NPs (60 nm in diameter) could cross mouse placenta and impair the development of mouse fetus [32]. Moreover, in a lung exposure mouse model, PS-NPs (20 nm in diameter) could translocate from mouse maternal lungs to placental and fetal tissues and thus disturb mouse fetal development [33]. These preliminary findings suggest that NPs may cross placental barrier and produce severe female reproductive toxicity.

### Nanoplastics and miscarriage

It has been estimated that 15-25% pregnant women worldwide may experience miscarriage [34]; and 1-5% might suffer from recurrent miscarriage (RM) [35]. In general, chromosomal abnormalities, uterine malformations, abnormal hormone levels, infections, psychological trauma, and immune system dysfunctions are considered as the causes or risk factors for miscarriage [36, 37]. However, approximately 50% miscarriage causes are still unexplained [38]. Notably, increasing evidence has demonstrated that environmental pollutants might act as important risk factors to induce miscarriage [39]. However, whether PS-NPs exposure might cause miscarriage has never been reported. It is also unclear whether PS particles are present in human villous tissues and their contents in villous tissues are associated with miscarriage.

### Nanoplastics and trophoblast cell apoptosis

Human trophoblast cells play essential roles in maintenance of normal pregnancy. Dysfunctions of human trophoblast cells might induce adverse pregnancy outcomes, such as miscarriage [39, 40]. Excessive apoptosis of human trophoblast cells is associated with the occurrence of miscarriage [41–43]. Moreover, trophoblast cells are very sensitive to environmental toxicants. It has been reported that triazole fungicide tebuconazole [44], Bisphenol A [45], heavy metal cadmium [46], and perfluorooctanoic acid [47] could induce trophoblast cell apoptosis. In our previous studies, we also found that benzo(a)pyrene (BaP) and benzo(a)pyrene-7,8-dihydrodiol-9,10-epoxide (BPDE) exposure could also induce trophoblast cell apoptosis [48, 49]. Emerging studies have shown that PS-NPs exposure induces oxidative stress, inflammation, mitochondrial dysfunctions, and apoptosis in neuronal cells, intestinal epithelial cells, neuroblastoma cells, and alveolar epithelial cells [50–54]. Moreover, mRNA sequencing analysis also suggests that PS-NPs exposure is related with cell apoptosis in human trophoblast cells [55] and in perfused human placenta [56]. Experimentally, PS-NPs can cross the placenta in mice and induce trophoblast cell apoptosis [57, 58]. However, the association between PS-NPs exposure and reproduction diseases are rarely explored. Whether PS-NPs exposure might cause trophoblast cell apoptosis and induce miscarriage is still elusive and should be fully investigated.

### Hypothesis and summary

In this study, we expect to explore whether PS plastics particles might be present in women villous tissues and whether PS-NPs exposure might cause human trophoblast cell apoptosis and then induce miscarriage. For this aim, we performed assays using RM and HC villous tissues, PS-NPs-exposed mouse miscarriage model, and PS-NPs-exposed human trophoblast cells. We found that PS plastic particles were present in human villous tissues and their contents were associated with miscarriage. Furthermore, PS-NPs exposure increased placental tissue (including trophoblast cells) apoptosis by activating Bcl-2/Caspase-2/Caspase-3 signaling through mitochondria pathway and eventually induced miscarriage. Supplement with Bcl-2 could efficiently reduce placental apoptosis and alleviate miscarriage in mouse model. Collectively, this study discovered the presence of PS plastics particles in women villous tissues, the new toxicological effects of PS-NPs, and the novel underlying mechanism, indicating that nanoplastics exposure is new risk factor for women unexplained miscarriage.

## Materials and methods

### Nanoplastics

Polystyrene-Nanoplastics (PS-NPs, 50 nm in diameter, DS50) and PS-NPs modified with fluorescein isothiocyanate (FITC-PS-NPs, 50 nm in diameter, GF50C, stable within five years) were purchased from Shanghai Huge Biotechnology Corporation. PS-NPs were suspended in ddH_2_O. PS-NPs were sealed and stored at 4 °C to avoid various contamination.

### Transmission election microscopy (TEM) image

The morphology and diameter of PS-NPs and FITC-PS-NPs were characterized by transmission electron microscopy (TEM), as the method described previously [59]. Trophoblast cells were exposed to 200 µg/mL PS-NPs for 24 h. After washing with PBS, the cells were fixed with 2.5% glutaraldehyde (pH = 7.4 at 25 ^o^C). After gradient dehydration with ethanol, the cells were then embedded in epoxy resin and cut into 60–80 nm sections. The sections were stained with uranyl acetate combined with lead citrate and visualized under a HITACHI HT7800/HT7700 transmission electron microscopy.

### Chemicals and reagents

Streptomycin, penicillin, and fetal bovine serum (FBS) were purchased from Hyclone (Logan, UT, USA). Ferrostatin-1 (Fer-1, HY-100,579), Necrostatin-1 (Nec-1, HY-15,760), Ac-FLTD-CMK (HY-111,675), Z-VAD-FMK (HY-16658B), Raptinal (HY-121,320), and Quinacrine dihydrochloride (HY-13,735 A) were purchased from MedChemExpress (Radnor, PA, USA). Mitochondrial Membrane Potential and Apoptosis Detection Kit was purchased from Beyotime (C1071S, Beyotime, CHN). Cell Counting Kit-8 (CCK8, ab228554), and reactive oxygen species (ROS) assay kit (ab113851) were purchased from Abcam (Cambridge, UK). Annexin V PE/7-AAD apoptosis detection kit was purchased from BD Biosciences (559,763, NJ, USA).

### Cell culture and PS-NPs-exposed trophoblast cells

Human trophoblast Swan 71 cells (passages 10–15) were cultured in Dulbecco’s modified Eagle’s medium (DMEM) (Hyclone, USA) supplemented with 10% FBS, 100 mg/mL penicillin, and 100 mg/mL streptomycin in an incubator with 5% CO_2_ at 37 °C. The suspension of PS-NPs was sonicated using a probe sonicator (6 mm probe; Branson Sonifier 250, Branson Ultrasonic Co., Danbury, CT, USA) at a power output of 13 W and frequency at 230 V/50 Hz for 5 min to avoid aggregation before each use [56]. Swan 71 cells were incubated in serum-free cell culture (without FBS) containing 0, 50, 100, 150, or 200 µg/mL PS-NPs for 24 h. The doses were selected based on the results reported in literature [55] and our own CCK8 assays and apoptosis assays in this study. NPs would adsorb proteins from cell culture medium to form protein corona on particles, which might affect the biological absorption, internalization, and toxicity of PS-NPs [60]. Therefore, serum-free cell culture was used in these assays to exclude the effects of proteins in cell culture.

### Internalization of FITC-PS-NPs into trophoblast cells

Internalization of FITC-PS-NPs into trophoblast Swan 71 cells was analyzed by flow cytometry, as described previously [61]. Briefly, 1 × 10^5^ cells in a 6-well plate were incubated with 5 µg/mL FITC-PS-NPs (50 nm in diameter) dispersed in serum-free medium at 37 °C for different time periods (0–24 h). Afterward, these cells were rinsed thrice with phosphate buffer saline (PBS, pH = 7.4 at 25 ^o^C) to remove FITC-PS-NPs outside cells and were then detached from plate with trypsin. Subsequently, these cells were washed thrice and re-suspended in 500 µL PBS. Cells containing FITC-PS-NPs were analyzed by flow cytometry on an Agilent instrument (ACEA NovoCyte, Agilent Biosciences). Data were analyzed with NovoExpress and Flowjo. The fluorescence intensity was recorded, with control cells as a reference.

### FITC-PS-NPs location in trophoblast cells

The location of FITC-PS-NPs in human trophoblast Swan 71 cells was measured on a confocal fluorescence microscopy. Briefly, Swan 71 cells in 6-well microscope slide were incubated in cell culture containing FITC-PS-NPs (5 µg/mL) for 1 h. Then, cells were treated with Nile Red (50 nM, HY-D0718, MedChemExpress, USA) and Hoechst 33,342 (0.5 mg/mL, C1025, Beyotime, CHN) for an additional 20 min, and then washed with PBS thrice to remove FITC-PS-NPs outside cells. Fluorescent images of Swan 71 cells containing FITC-PS-NPs were accorded on a confocal laser scanning microscope (Zeiss LSM 880, Wetzlar, Germany).

### Cell viability

Swan 71 cells (3 × 10^3^ cells) were treated with PS-NPs at different concentrations (0, 50, 100, 150, or 200 µg/mL) for 24 h. Afterward, cells were re-cultured in a fresh medium containing 10% CCK-8 reagent for 1 h before the determination of the optical absorbance at 450 nm. Cell viability = (absorbance of experimental group - absorbance of blank) / (absorbance of control group - absorbance of blank).

### Apoptosis of trophoblast cells

Swan 71 cells were seeded in a 6-well plate for 24 h, washed twice with cold PBS, and then re-suspended in 500 µL binding buffer. Then, the cells were incubated with PE Annexin V (5 µL) and 7-AAD (5 µL) for 15 min in dark. The apoptosis rates (including early and late apoptosis) of Swan 71 cells were analyzed by flow cytometry (ACEA NovoCyte, Agilent Biosciences) within 24 h.

### Apoptosis levels in tissues

The levels of apoptosis in women villous tissues and mouse placental tissues were evaluated using terminal deoxynucleotidyl transferase-mediated deoxyuridine triphosphate (dUTP) nick end labeling (TUNEL) assay kit (C1089, Beyotime) according to the manufacturer’s protocols. Briefly, human villous tissues (*n* = 12 in both groups) and mouse placental tissues (*n* = 9 in each group) were made into 3-µm-thick paraffin sections. Paraffin sections were deparaffinized in dimethylbenzene and rinsed in gradient ethanol to water. The slides were incubated in proteinase K (20 µg/mL) at 37 °C for 20 min. After rinsing with PBS thrice, the slides were incubated with terminal deoxynucleotidyl transferase enzyme and Cy3-labeled dUTP mixture at 37 °C for 1 h in dark. The levels of apoptosis in tissues were examined using confocal laser scanning microscope (Zeiss LSM 880, Wetzlar, Germany).

### Mitochondrial membrane potential (MMP)

The MMP of Swan 71 cells was assessed using Mitochondrial Membrane Potential and Apoptosis Detection Kit with Mito-Tracker Red CMXRos (C1071S, Beyotime, CHN), according to the manufacturer’s protocols. Briefly, Swan 71 cells were incubated with Mito Tracker Red CMXRos dye solution at room temperature for 20 min in dark. The MMP was determined on a fluorescence inverted microscope (Zeiss Axio Observer, Wetzlar, Germany). The red fluorescence of Mito Tracker Red CMXRos indicated the changes in MMP, which was recorded and quantified using ImageJ.

### Measurement of ROS

Intracellular reactive oxygen species (ROS) in trophoblast Swan 71 cells was measured using ROS Assay Kit (Abcam, ab113851). Briefly, Swan 71 cells were exposed to PS-NPs (0, 100, or 200 µg/mL) for 1 or 2 h, washed with PBS, and then incubated with 1 mM dichlorodihydrofuorescein diacetate (DCFH-DA) at 37 °C for 30 min in dark. Then, cells were washed with serum-free culture thrice to remove remaining DCFH-DA probe outside cells. The levels of ROS in women villous tissues and mouse placental tissues were evaluated using ROS Assay Kit (BB-470,522, BestBio) according to the standard protocols. Briefly, human villous tissues (*n* = 12 in both groups) and mouse placental tissues (*n* = 9 in each group) were made into 3-µm-thick paraffin sections were stained with BBoxiProbe® O11 ROS Probe for 30 min in dark. The images were captured on a fluorescence inverted microscope (Zeiss Axio Observer, Wetzlar, Germany). The fluorescence intensity was analyzed using software ImageJ.

### RNA isolation and RT-qPCR analysis

Total RNAs in Swan 71 cells, villous tissues, or mouse placental tissues were isolated using Trizol reagent (Thermo Fisher Scientific, Waltham, USA) and their concentrations were measured using a Nanodrop 2000 (Thermo Fisher Scientific, Waltham, USA). Reverse transcription was performed using the First Strand cDNA Synthesis Kit (Takara, Kyoto, Japan). RNA expression levels were analyzed using the SYBR Green dye (Takara, Kyoto, Japan) on CFX96 system (Bio-Rad, CA, USA). The relative expression levels were calculated using 2^−ΔΔCt^ method with GAPDH mRNA as the endogenous control. The specific primers were synthesized by Thermo Fisher Scientific Company and their sequences were shown in Table S1.

### Cell transfection

Bcl-2 was silenced by transfecting trophoblast cells with its two distinct siRNAs (si-Bcl-2#1 and si-Bcl-2#2), customized from Thermo Fisher Scientific Company. Bcl-2 was also overexpressed by transfecting trophoblast cells with its overexpression plasmid (pcDNA3.1-Bcl-2), which was customized from Thermo Fisher Scientific Company. Swan 71 cells (3 × 10^5^ cells/well) were transfected with si-Bcl-2 or pcDNA3.1-Bcl-2 for 24 h in Lipofectamine 3000 (Life Technologies, Gaithersburg, USA) according to the manufacturer’s protocols. The sequences of siRNAs and pcDNA3.1-Bcl-2 were shown in Table S2 and S3. Western blotting and RT-qPCR were used to validate their transfection efficiencies.

### Western blot analysis

Total proteins were extracted from Swan 71 cells, villous tissues, or mouse placental tissues with RIPA lysis buffer on ice for 30 min. Protein concentrations were determined using BCA Protein Quantification Kit (Vazyme, Nanjing, China). Western blotting analysis was performed as described previously [62]. Primary antibodies included anti-β-actin (ab8226, 1:5000), anti-β-Tubulin (ab108342, 1:10000), anti-GAPDH (ab8245,1: 5000), anti-Caspase-3 (ab184787,1:1000), anti-Bcl-2 (ab182858, 1:1000), and anti-Caspase-2 (ab179520, 1:2500), all of which were purchased from Abcam (Cambridge, UK). Secondary antibodies included goat anti-rabbit immunoglobulin G (IgG) (dilution 1:10,000; ab207995, Abcam) and goat anti-mouse IgG (dilution 1:10,000; ab207996, Abcam). The intensities of Western blotting bands were quantified using Image J software with β-actin, Tubulin, or GAPDH as loading control. In some cases, proteins with different molecule weights were analyzed in one gel and one membrane. Subsequently, this membrane was cut into different parts based on the locations of protein bands and then stained with their corresponding antibody. Loading controls with different molecular weights from the target proteins were selected to avoid overlap or proximity. For clarity, only one loading control was shown in figures.

### Tissues collection and statement

Villous tissue samples were collected from 18 patients with unexplained recurrent miscarriage (RM group) and 18 women who had elective miscarriage to terminate unwanted pregnancies (healthy control (HC) group) between 25 and 30 years old, as the methods described previously [42, 62, 63]. Any woman with one of the following features was excluded, including uterine abnormalities, abnormal karyotype, autoimmune abnormality, antiphospholipid antibody syndrome, endocrine or metabolic diseases, polycystic ovarian syndrome, pre-eclampsia or eclampsia, as described previously [42, 62, 63]. All instruments, including metal tweezers, metal scalpels, and metal clippers, were washed using ddH_2_O (18 MΩ). After collection and washing with sterile saline, villous tissues were stored in cooled glass containers with wooden lids, avoiding contact with plastic material. An aliquot of tissue samples were transported to laboratory for Py-GC/MS analysis; and the rest of samples were stored at -80 ^o^C for subsequent protein and RNA extraction. The protocols were approved by the Ethics Committee of the Eighth Affiliated Hospital, Sun Yat-sen University. Written informed consents were collected from all the participants before enrollment.

### Py-GC/MS analysis of PS plastic particles in miscarriage villous tissues

PS plastic particles in villous tissues were analyzed by Py-GC/MS, as the methods described previously [64, 65]. To avoid plastic contamination, non-plastic instruments and tubes were used in sample collection, storage, processing, and analysis. Villous samples were stored in glass tubes with pre-cleaned stainless steel trays. Cotton laboratory coats, face masks, headgear, and single-use latex gloves were worn during entire experiments. Villous tissue samples (2.0 g) were rinsed with PBS and lately digested in 10% KOH solution at room temperature for 48 h. All solutions were filtered by 0.22 μm fiberglass membrane. Then, the mixture was heated on a graphite plate at 110 °C for 2 h and dried in blast oven at 110 °C. The combined PBS and 10% KOH solution was used as blank control, which was processed similarly during sample collection, storage, processing, and analysis. Afterward, all these samples were analyzed by pyrolysis-gas chromatography/mass spectrometry (Py-GC/MS, Py-3030D) on gas chromatography (GC 2010, Shimadzu, Rtx-5 ms column with 30 m × 0.25 mm × 0.25 μm) coupled with mass spectrometry (MS-QP 2010, Shimadzu). The temperature program for GC was set as the following: at 40 °C for 2 min, increased to 230 °C at 20 °C/min, then increased to 320 °C at 50 °C/min, and at 320 °C for 14 min. Helium (purity 5.0) was used as carrier gas with a flow rate of 1 mL/min. The recovery rates of PS particles in tissue samples were more than 90%. PS fragments with a limited mass/charge ratio range were detected using SIM (Selected Ion Monitoring) acquisition mode [65]. The presence of PS plastics particles was identified by the characteristic peaks at m/z of 51, 78, and 104 corresponded to the specific fragments of styrene monomer, and the characteristic peaks at m/z of 91 and 312, corresponding to the specific fragments of polystyrene trimer, as described previously [64]. In blank control, no such characteristic peaks were detected.

### PS-NPs-exposed mouse miscarriage model

PS-NPs-exposed mouse model was constructed as the method described previously [66, 67]. Briefly, C57BL/6 mice (Charles River Company, 6-8-week-old) were housed under standard environmental conditions (12 h light/dark cycle, 22 ℃), during which time they received standard chow and tap water ad libitum. Female mice were mated with male mice overnight, and the appearance of vaginal plug was considered as the first day of pregnancy (D1), which was further validated by monitoring the increase in weight. Exposure of mice to 50 mg/kg/d 100 nm PS-NPs for 17 days shows that PS-NPs can enter placenta and induced excess ROS and apoptosis in mouse placenta [52]. Meanwhile, exposure of mice to 30 mg/kg/d 0.8 μm PS-MPs for 35 days induced inflammation of ovaries and also reduced the quality of oocytes [28]. In this study, we selected smaller 50 nm PS-NPs and a wider exposure dose (0, 25, 50, 100 mg/kg/d) in mouse assays to explore the reproductive toxicity. These pregnant mice were randomly divided into two large groups (PS-NPs treatment group and Bcl-2 treatment group). PS-NPs treatment group (each *n* = 9): ① control, ② 25 mg/kg PS-NPs, ③ 50 mg/kg PS-NPs, ④ 100 mg/kg PS-NPs. Bcl-2 treatment group (each *n* = 9): ① 100 mg/kg PS-NPs + pcDNA3.1, ② 100 mg/kg PS-NPs + pcDNA3.1-Bcl-2 (murine Rock1). They were given 0, 25, 50, or 100 mg/kg PS-NPs in water through oral gavage from D1 to D14. Meanwhile, PS-NPs-exposed pregnant mice (Bcl-2 treatment group) were also intraperitoneally injected with empty vector pcDNA3.1 or pcDNA3.1-BCl-2 once per three days. The sequences of pcDNA3.1-Bcl-2 were shown in Table S3. All mice were weighed daily, and they were euthanized by injection with Nembutal (100 mg/kg) on D14 to collect uteri. All placenta was collected from every mouse in each group. The placental tissues were manually dissected, snap-frozen in liquid nitrogen, and then stored at -80 ℃ for subsequent RT-qPCR and Western blotting analysis. The protocol was approved by the Medical Ethics Committee of the Eighth Affiliated Hospital, Sun Yat-sen University.

### Statistical analysis

All experiments were replicated thrice independently with similar results. The data were presented as mean ± SD (standard deviation, *n* = 3). Statistical analysis was performed using GraphPad Prism 8.0 (GraphPad Inc, USA). The correlation analysis of the relative expression levels was performed using Pearson analysis. Receiver Operating Characteristic (ROC) curves were plotted using survival ROC package. Area Under Curve (AUC) was calculated as the area under the ROC curve. Mann-whitney test was used to analyze the differences between two groups with non-normal distribution. Fisher’s exact test was used to analyze categorical variables. Logistic regression was used to analyze the odds ratio (OR) of each variable and the risk of unexplained miscarriage and its 95% confidence interval (95% CI). The Student’s t-test and one-way ANOVA followed by Dunnett’s test were used to evaluate the differences between two groups and among three or more than three groups, respectively. *P* < 0.05 was considered statistically significant.

## Results

### PS plastic fragments were detected out in RM villous tissues and its high contents were associated with miscarriage

**Fig. 1:**
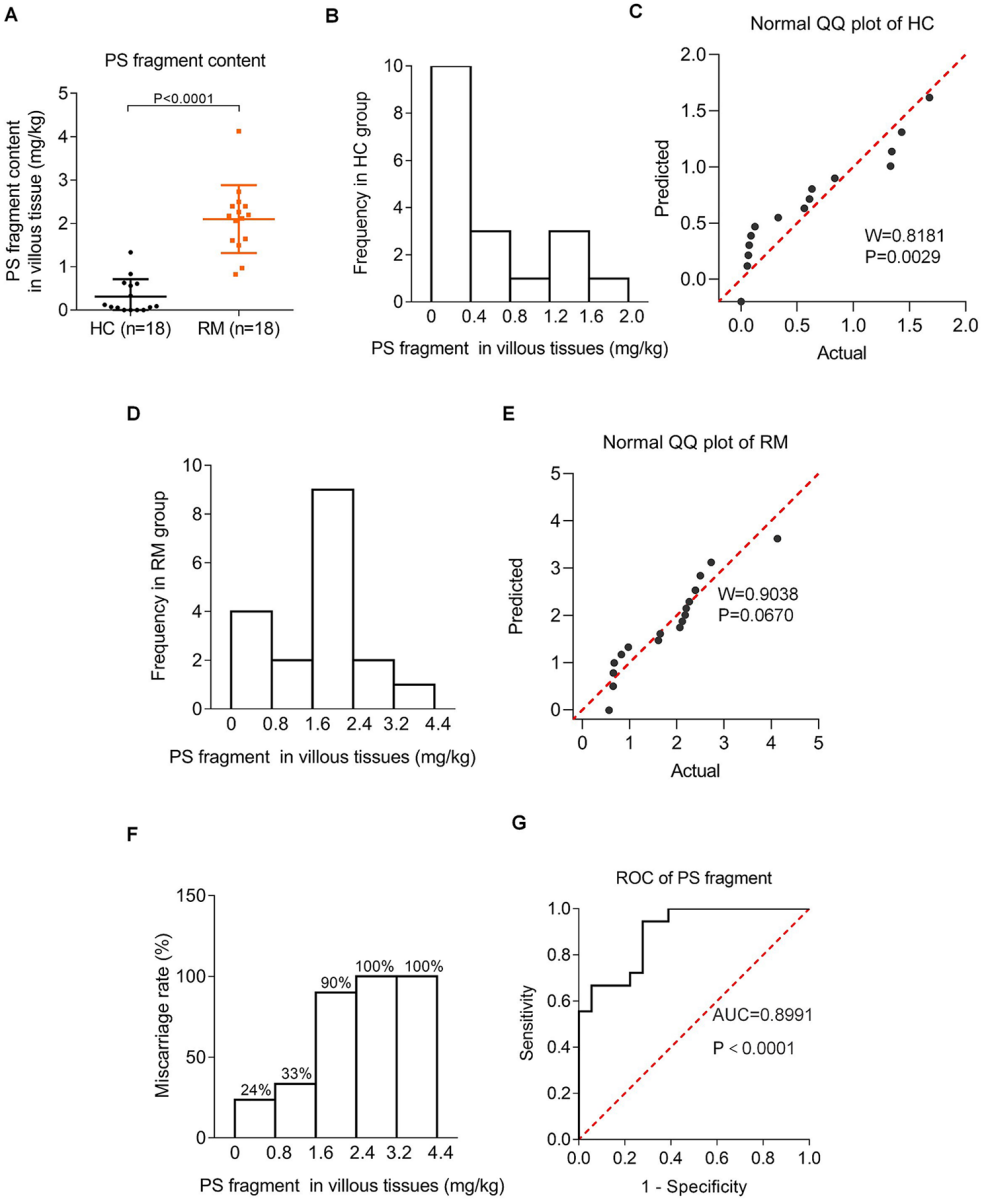
PS plastic fragments were detected out in human villous tissues and their high contents were positively associated with miscarriage. (**A**) The contents of PS plastic fragment in HC and RM villous tissues analyzed by Py-GC/MS (each *n* = 18). (**B**) The histogram of PS plastic fragment frequency in HC villous tissues. (**C**) The normal QQ plot of PS fragment content in HC villous tissues. (**D**) The histogram of PS fragment frequency in RM villous tissues. (**E**) The normal QQ plot of PS fragment content in RM villous tissues. (**F**) The ratios of RM women in total women in each range of the contents of PS plastic fragments. (**G**) The ROC curve of the diagnostic value of PS fragment contents for RM

To explore the potential adverse effects of PS plastics particles on miscarriage, firstly, we detected whether PS plastic fragments might be present in villous tissues of miscarriage women and whether their contents might be associated with miscarriage. For this aim, we collected villous tissue samples from unexplained recurrent miscarriage (RM) patients and their matched healthy control (HC) group (*n* = 18), as the methods described previously [42, 62, 63]. The known causes, such as chromosomal abnormalities, hormonal abnormality and uterine deformation, have been excluded [29, 68]. The parameters (such as maternal age, pre-pregnancy BMI, gestational days) of these two groups did not show significant differences (Table S4). During sample collection, storage, and analysis, non-plastic instruments and tubes were used to avoid the plastic contaminants. Then, PS plastic fragments were separated from villous tissue samples and were analyzed by Py-GC/MS, in which PS plastic fragments were completely degraded, separated by GC, and analyzed by MS [69]. PS plastic fragments were detected out from RM and HC villous tissues (each *n* = 18) but not from the blank control. The contents of PS fragments were in the range from 0 to 1.68 mg/kg with median (95% confidence interval (CI)) of 0.227 (0.056–0.834) mg/kg in HC group and in the range from 0.56 to 4.13 mg/kg with median (95% CI) of 2.09 (0.826, 2.394) mg/kg in RM group (Fig. 1A; Table 1). The contents of PS fragments were significantly higher in RM vs. HC villous tissues. The distribution of PS fragments followed a skewed distribution in HC villous tissues **(**Fig. 1B, C**)** and a normal distribution in RM villous tissues (Fig. 1D, E**)**. Then, we divided the contents of PS fragments into several ranges and counted the numbers of RM and HC women in every range. The ratios of RM women in total women showed an increasing trend with increasing the contents of PS fragments **(**Fig. 1F**)**, implying that the contents of PS fragments were positively associated with miscarriage. This was further confirmed by logistic regression analysis with the odds ratio (OR) of 34 and corresponding 95% CI of 3.61 to 320 **(**Table 2**)**, indicating that PS plastic fragments in villous tissues were risk factors for miscarriage. Receiver Operating Characteristic (ROC) curve analysis showed that the Area Under Curve (AUC) value of the contents of PS fragments within 95% confidence interval was 0.899 **(**Fig. 1G**)**, indicating that the contents of PS fragments in villous tissues had good predicative capability for miscarriage with high specificity and sensitivity. Taken together, these results confirmed that PS plastic fragments were present in human villous tissues, their contents were higher in RM vs. HC group, and their contents in villous tissues were positively associated with miscarriage.

**Table 1:** Mann-whitney test analysis of PS plastic fragment contents in HC and RM villous tissues (*n* = 18)

PS fragments in villous tissues	HC (*n* = 18)	RM (*n* = 18)
Median (95% CI)	0.227 (0.056, 0.834)	2.09 (0.826, 2.39)
Min-max	0–1.68	0.564–4.13
*P*_10_-*P*_90_ (Reference range)	0–1.46	-
P value	*P* < 0.0001	

CI: Confidence interval; PS: Polystyrene; *P*: Percentile; P value: Mann-whitney test

**Table 2:** Fisher’s exact test and logistic regression analysis of PS plastic fragment contents in HC and RM villous tissues (*n* = 18)

PS fragment in villous tissues	HC (*n* = 18)	RM (*n* = 18)	Total	P value	OR	95% CI
*P*_10_-*P*_90_ (Reference range)	17	6	23	< 0.0001	34	3.61 to 320
> 1.46	1	12	13			
Total	18	18	36			

OR: Odd Ratio; P value: Fisher’s exact test

### PS-NPs exposure induced mouse miscarriage

**Fig. 2:**
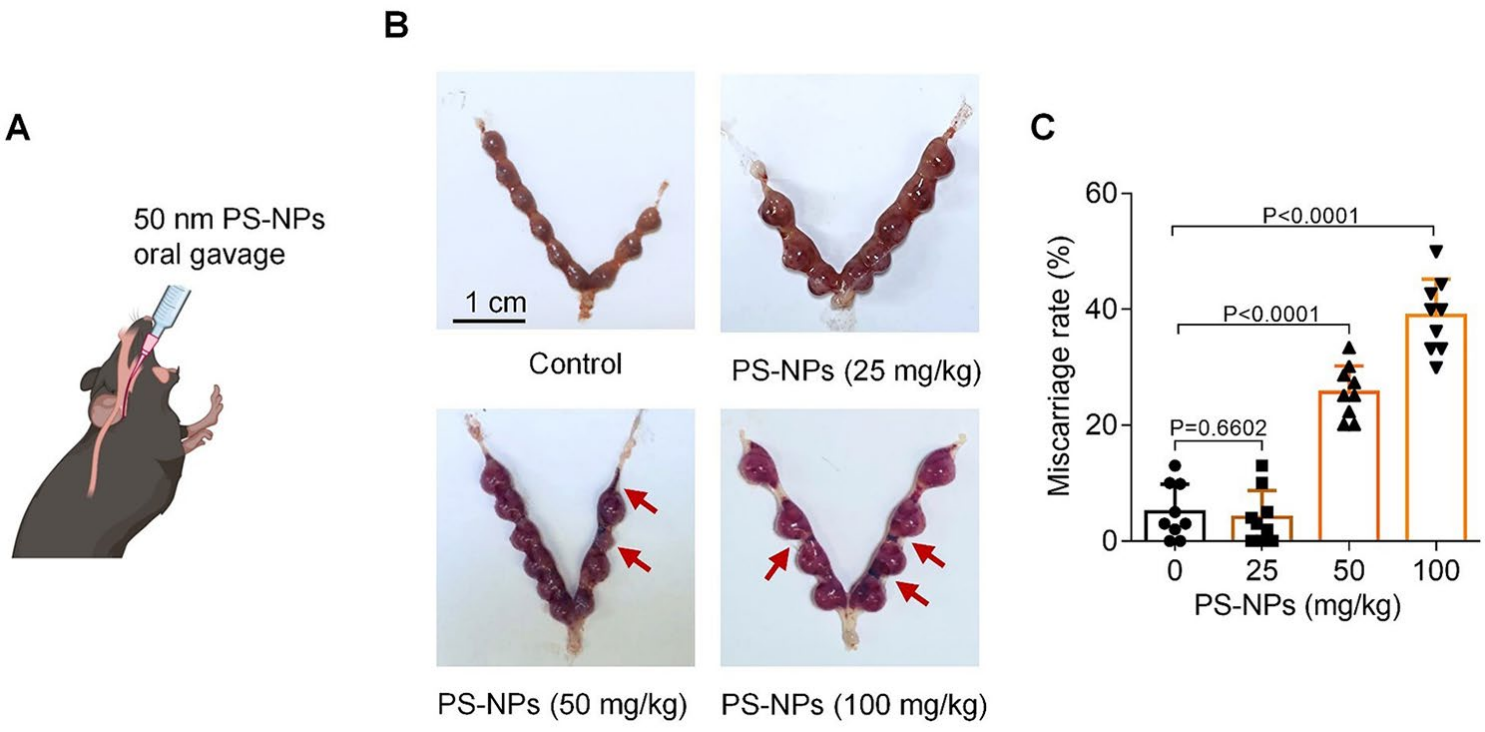
PS-NPs exposure induced mouse miscarriage. (**A**) Schematic diagram of mouse model exposed to 50 nm PS-NPs by oral gavage. (**B**, **C**) The embryo adsorption and miscarriage rates in 0, 25, 50, or 100 mg/kg PS-NPs-exposed pregnant mice (each *n* = 9, scale bar, 1 cm)

To directly examine whether PS-NPs exposure might induce miscarriage, we treated pregnant mice with varying doses (0, 25, 50, or 100 mg/kg) of PS-NPs by oral gavage and detected mouse miscarriage rates (Fig. 2A). Exposure to saline or 25 mg/kg PS-NPs did not show obvious embryo adsorption; but exposure to 50 and 100 mg/kg PS-NPs showed embryo adsorption, as indicated by the smaller or darker appearance relative to the healthy embryos [42] (Fig. 2B). The miscarriage rates were calculated as the number of embryo resorption divided by the number of total embryos in one mouse. The average miscarriage rates were increased with increasing the doses of PS-NPs (Fig. 2C). Taken together, these assays confirmed that exposure of pregnant mice to PS-NPs could induce miscarriage.

### The characterization and internalization of PS-NPs in human trophoblast cells

**Fig. 3:**
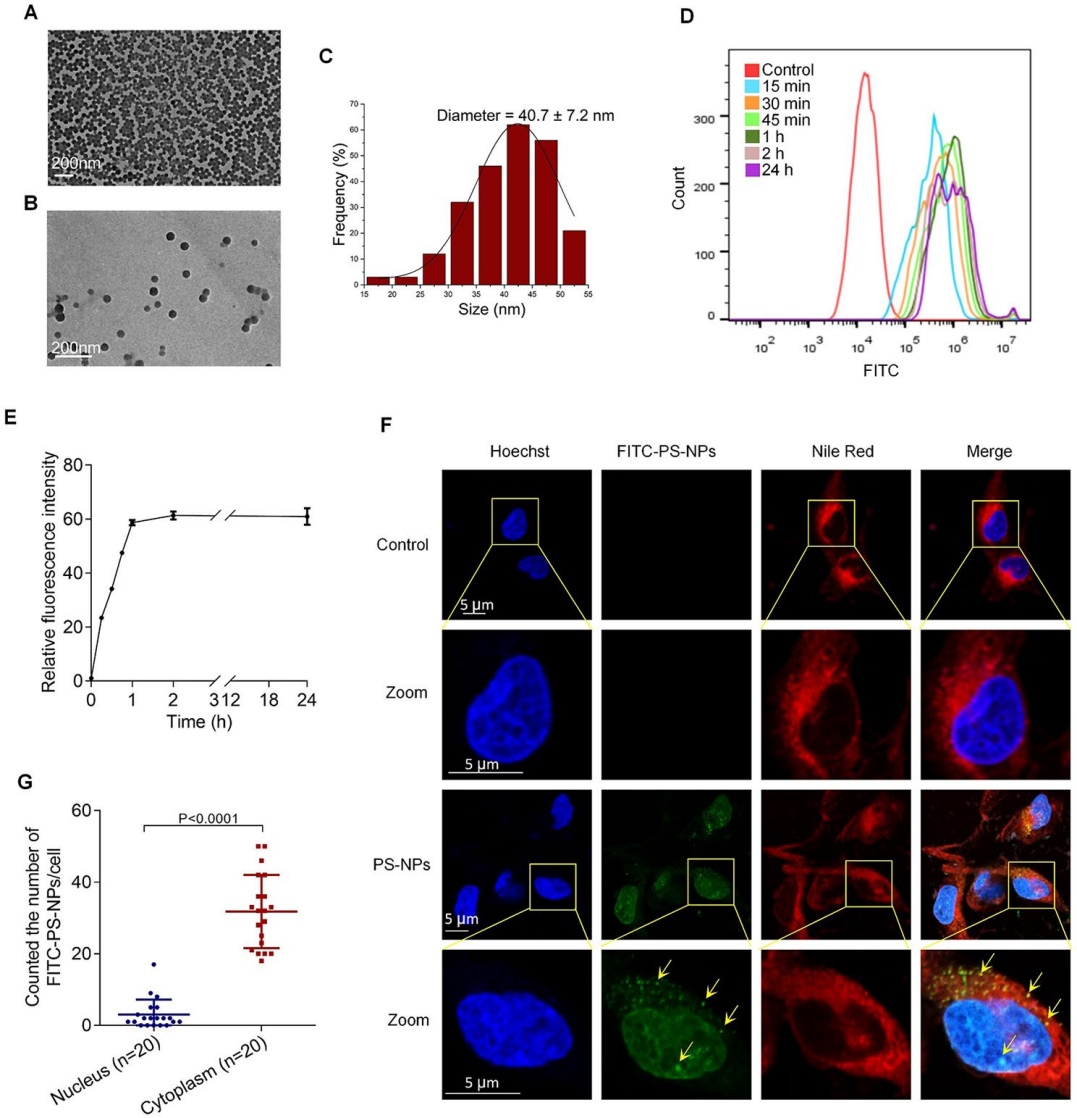
The characterization and internalization of PS-NPs in human trophoblast cells. (**A**, **B**) TEM image of PS-NPs (scale bar, 200 nm). (**C**) Size analysis of PS-NPs in TEM images. (**D**, **E**) Internalization of FITC-PS-NPs into Swan 71 cells analyzed by flow cytometry. (**F**) Distribution of FITC-PS-NPs in Swan71 cells detected by confocal microscopy. Nucleus (blue), cytoplasm (red), and FITC-PS-NPs (green, indicated by yellow arrows). Scale bar, 5 μm for all images. (**G**) The average number of FITC-PS-NPs were counted in the nucleus and cytoplasm of Swan 71 cells

Trophoblast cells in villous tissues take important roles in healthy pregnancy. Dysfunctions of trophoblast cells might induce miscarriage. Then, we selected trophoblast Swan 71 cells as cell model to study the adverse effects of PS-NPs on trophoblast cell functions. Firstly, transmission electron microscope (TEM) analysis showed that PS-NPs after ultrasonication were highly dispersed with uniform and spherical morphology (Fig. 3A, B). The average diameter of PS-NPs was 41 ± 7 nm (mean ± SD) (Fig. 3C). It has been reported that PS-NPs could cross placental barrier [33]. Thus, we detected whether PS-NPs might be internalized into Swan 71 cells. To evaluate this, we used fluorescein isothiocyanate-labeled PS-NPs (FITC-PS-NPs, 50 nm, 5 µg/mL) as the substitutes of PS-NPs. Flow cytometry analysis showed that FITC-PS-NPs could be easily internalized into Swan 71 cells within 1 h and kept constant at the maximal level for subsequent at least 24 h (Fig. 3D, E). Confocal microscopy analysis further showed that FITC-PS-NPs (5 µg/mL) were primarily localized in trophoblast cell cytoplasm **(**Fig. 3F, G**)**. Based on the fluorescence intensity of FITC-PS-NPs, we counted the average number of FITC-PS-NPs in the nucleus and cytoplasm as approximately 3 ± 4 and 32 ± 10 (mean ± standard deviation (SD)), respectively. Taken together, these results demonstrated that PS-NPs could be easily internalized into trophoblast cells and were primarily distributed in trophoblast cell cytoplasm at a relatively constant concentration.

### PS-NPs exposure induced human trophoblast cell apoptosis

**Fig. 4:**
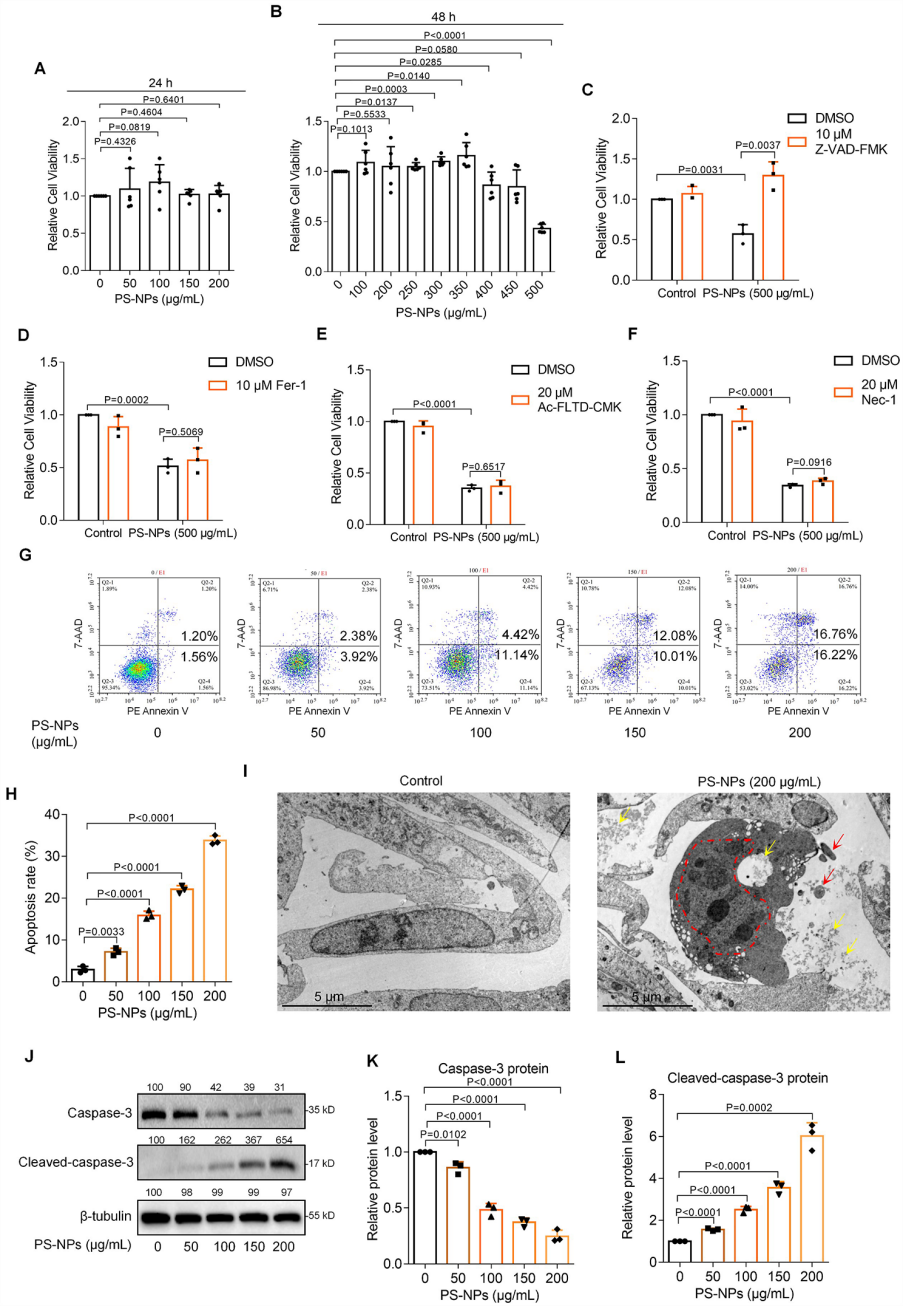
PS-NPs exposure induced human trophoblast cell apoptosis. (**A**) Cell viability of Swan 71 cells exposed to 0-200 µg/mL PS-NPs for 24 h. (**B**) Cell viability of Swan 71 cells exposed to 0-500 µg/mL PS-NPs for 48 h. (**C**-**F**) Cell viability of 500 µg/mL PS-NPs-exposed Swan 71 cells treated with apoptosis inhibitor cystatin Z-VAD-FMK (**C**), ferroptosis inhibitor Fer-1 (**D**), pyroptosis inhibitor Ac-FLTD-CMK (**E**), or necrosis inhibitor Nec-1 (**F**) for 48 h. (**G**, **H**) Flow cytometry analysis and their quantification of apoptosis rates (total early and late apoptosis) in 0-200 µg/mL PS-NPs-exposed Swan 71 cells for 24 h. (**I**) TEM image of 0 or 200 µg/mL PS-NPs-exposed Swan 71 cells (scale bar, 5 μm; PS-NPs were indicated by yellow arrows; cell pyknosis, nuclear membrane wrinkling, chromatin aggregation, division, and edge shift were indicated by red dashed line; apoptotic bodies were indicated by red arrows). (**J**-**L**) Western blot analysis and the relative quantification of the protein levels of Caspase-3 and Cleaved-caspase-3 in 0-200 µg/mL PS-NPs-exposed Swan 71 cells for 24 h

Subsequently, we tested the potential adverse effects of PS-NPs on human trophoblast cell functions by treating Swan 71 cells with varying concentrations (0, 50, 100, 150, or 200 µg/mL) of PS-NPs. Trophoblast cell viability was almost unchanged with exposure to 0-200 µg/mL PS-NPs for 24 h but was significantly decreased with exposure to 400, 450, or 500 µg/mL PS-NPs for 48 h **(**Fig. 4A, B**)**, suggesting that higher concentrations of PS-NPs could reduce trophoblast cell viability. To distinguish which cell death mode was significantly regulated, we treated PS-NPs-exposed trophoblast cells with several specific inhibitors, including apoptosis inhibitor cystatin Z-VAD-FMK, ferroptosis inhibitor ferrostatin-1 (Fer-1), pyroptosis inhibitor Ac-FLTD-CMK, or necrosis inhibitor Necrosstatin-1 (Nec-1). We found that treatment with apoptosis inhibitor **(**Fig. 4C**)**, but not other inhibitors **(**Fig. 4D-F**)**, could efficiently rescue PS-NPs-induced trophoblast cell death, indicating that PS-NPs primarily induced trophoblast cell apoptosis. To focus the effects of PS-NPs on trophoblast cell apoptosis, we also treated trophoblast cells with apoptosis promoter Raptinal [70] or Quinacrine dihydrochloride [71]. Treatment with Raptinal, Quinacrine dihydrochloride, or PS-NPs reduced trophoblast cell viability, and co-treatment with Z-VAD-FMK could well rescue trophoblast cell viability (Fig. S1).

Subsequently, flow cytometry analysis showed that the levels of trophoblast cell apoptosis (including early apoptosis and late apoptosis) were increased with increasing PS-NPs concentrations (0-200 µg/mL for 24 h) in a dose-dependent manner (Fig. 4G, H). Cell apoptosis and viability were analyzed by determining phosphotidylserine on the cell membrane and the amount of dehydrogenases released from cells, respectively [72, 73], which might possibly show different sensitivity to the doses of PS-NPs. TEM images showed that PS-NPs were present in PS-NPs-exposed Swan 71 cells (indicated by yellow arrows) and this exposure resulted in cell pyknosis, nuclear membrane wrinkling, chromatin aggregation, division, and edge shift (red dashed line), and also induced the formation of apoptotic bodies (red arrows) (Fig. 4I). Moreover, PS-NPs exposure also reduced Capase-3 protein levels and increased Cleaved-capase-3 protein levels, two typical indicators for cell apoptosis, in a dose-dependent manner **(**Fig. 4J-L**)**. All these results confirmed that PS-NPs exposure induced trophoblast cell apoptosis.

### PS-NPs induced trophoblast cell apoptosis through mitochondrial Bcl-2/caspase-2/caspase-3 pathway

**Fig. 5:**
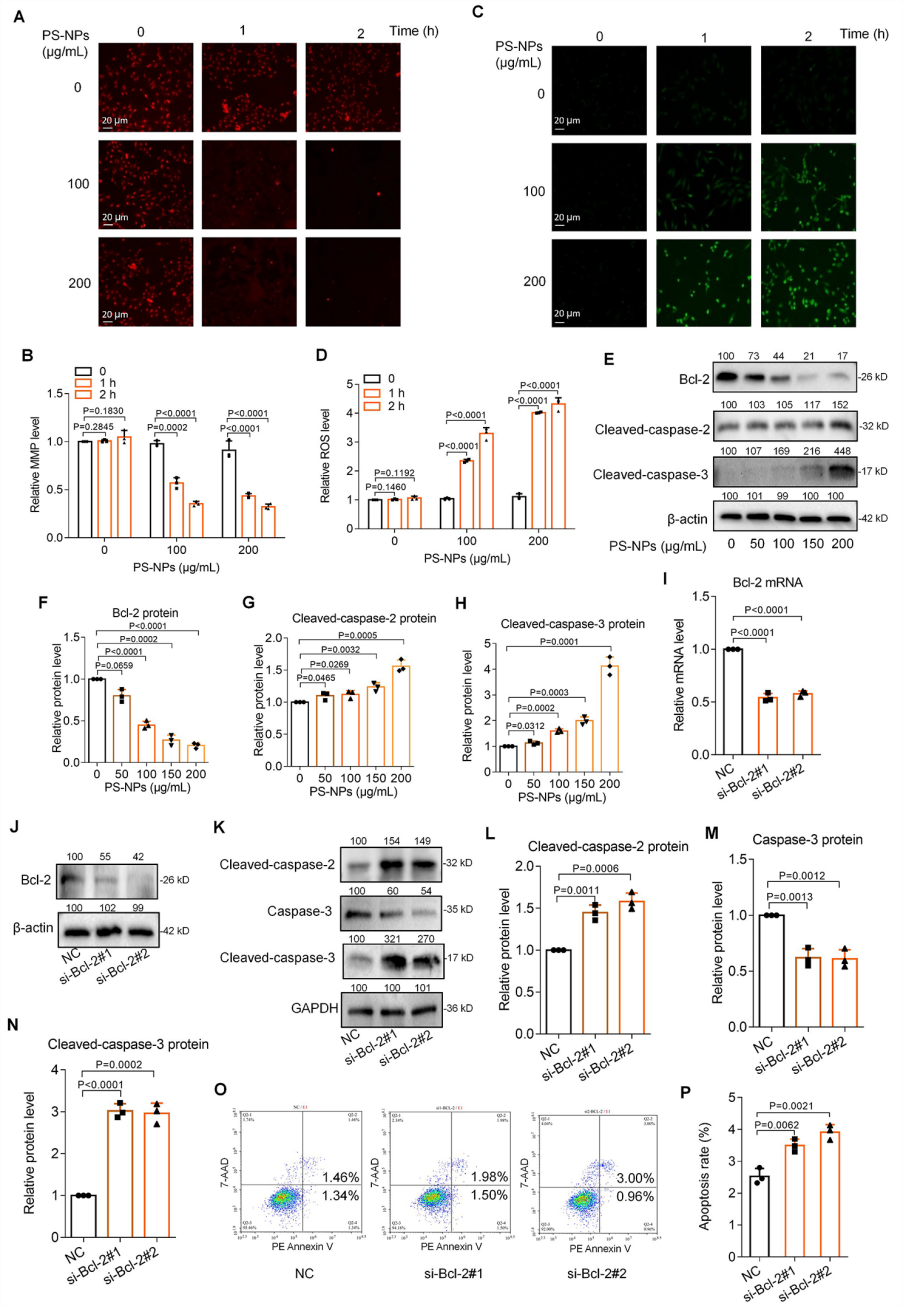
PS-NPs induced trophoblast cell apoptosis through mitochondrial Bcl-2/Caspase-2/Caspase-3 pathway. (**A**, **B**) MMP levels in Swan 71 cells exposed to 0, 100, or 200 µg/mL PS-NPs for 0, 1, or 2 h (scale bar, 20 μm). (**C**, **D**) ROS levels in Swan 71 cells exposed to 0, 100, or 200 µg/mL PS-NPs for 0, 1, or 2 h (scale bar, 20 μm). (**E**-**H**) The protein levels of Bcl-2, Cleaved-caspase-2, and Cleaved-caspase-3 in Swan 71 cells exposed to 0, 50, 100, 150, or 200 µg/mL PS-NPs for 24 h. (**I**, **J**) The mRNA and protein levels of Bcl-2 in trophoblast cells with Bcl-2 knockdown. (**K**-**N**) The protein levels of Cleaved-caspase-2, Caspase-3, and Cleaved-caspase-3 in trophoblast cells with Bcl-2 knockdown. (**O**, **P**) Flow cytometry analysis and their quantification of apoptosis rates (total early and late apoptosis) of trophoblast cells with Bcl-2 knockdown

Subsequently, we explored the potential mechanism how PS-NPs induced trophoblast cell apoptosis. Apoptosis can be triggered by two distinct pathways: the intrinsic (mitochondrial or Bcl-2) pathway and the extrinsic (death receptor) pathway [74]. Since mitochondria are important bioenergy sources and biosynthetic centers in cells and are also sensitive to environmental toxicants, we preferentially tested whether PS-NPs exposure might induce trophoblast cell apoptosis through mitochondrial pathway. The levels of mitochondrial membrane potential (MMP) were decreased **(**Fig. 5A, B**)** and the levels of ROS were increased **(**Fig. 5C, D**)** in PS-NPs-exposed Swan 71 cells in a dose- or time-dependent manner. Moreover, the protein levels of anti-apoptotic Bcl-2 were decreased and those of apoptotic Cleaved-caspase-2 and Cleaved-caspase-3 were increased in PS-NPs-exposed Swan 71 cells **(**Fig. 5E-H**)**, all of which contributed to mitochondrial apoptosis. Therefore, PS-NPs exposure increased mitochondrial permeability, produced excessive ROS, and induced trophoblast cell apoptosis through mitochondrial pathway.

To explore whether Bcl-2 might regulate the expression levels of Cleaved-caspase-2 and Cleaved-caspase-3, we silenced Bcl-2 in human trophoblast cells by transfecting with its two distinct siRNAs (Fig. 5I, J). Knockdown of Bcl-2 up-regulated the protein levels of Cleaved-caspase-2 and Cleaved-caspase-3 **(**Fig. 5K-N**)**. Meanwhile, flow cytometry analysis also showed that knockdown of Bcl-2 increased Swan 71 cell apoptosis **(**Fig. 5O, P**)**. Therefore, these results indicated that PS-NPs induced trophoblast cell apoptosis through mitochondrial pathway by activating Bcl-2/Caspase-2/Caspase-3 signaling pathway.

### Supplement with Bcl-2 alleviated apoptosis in PS-NPs-exposed human trophoblast cells

**Fig. 6:**
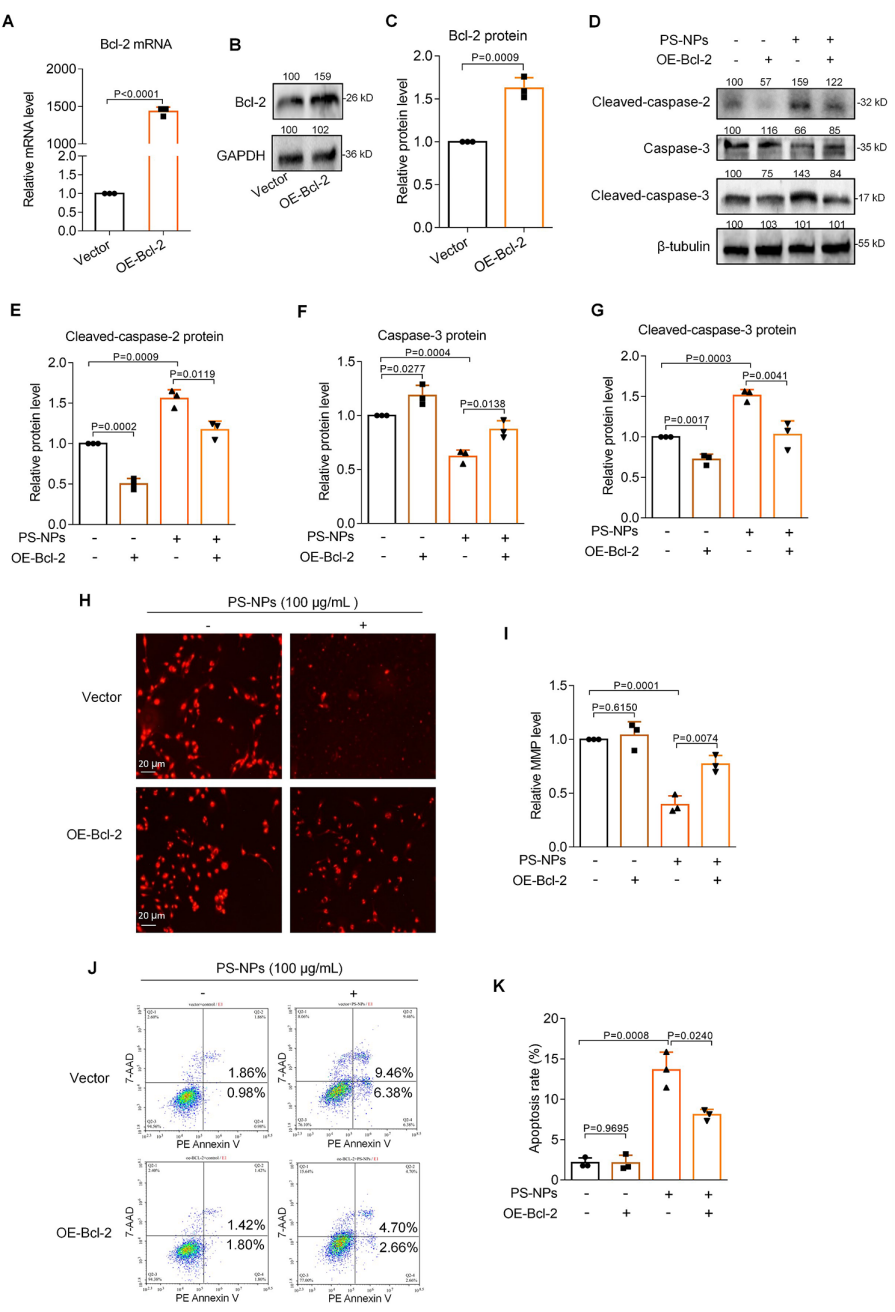
Supplement with Bcl-2 alleviated apoptosis in PS-NPs-exposed human trophoblast cells. (**A**-**C**) The mRNA and protein levels of Bcl-2 in Swan 71 cells with Bcl-2 overexpression. (**D**-**G**) The protein levels of Cleaved-caspase-2, Caspase-3, and Cleaved-caspase-3 in trophoblast cells exposed to 0 or 100 µg/mL PS-NPs and transfected with or without Bcl-2 overexpression plasmid for 24 h. (**H**, **I**) MMP levels in trophoblast cells exposed to 0 or 100 µg/mL PS-NPs and transfected with or without Bcl-2 overexpression plasmid for 1 h (scale bar, 20 μm). (**J**, **K**) Flow cytometry analysis and quantification of apoptosis rates (total early and late apoptosis) in trophoblast cells exposed to 0 or 100 µg/mL PS-NPs and transfected with or without Bcl-2 overexpression plasmid for 24 h

Since PS-NPs induced human trophoblast cell apoptosis through down-regulating Bcl-2 protein levels, we wondered whether supplement with Bcl-2 could alleviate apoptosis in PS-NPs-exposed trophoblast cells. For this aim, Bcl-2 was overexpressed in Swan 71 cells by transfecting with its overexpression plasmid **(**Fig. 6A-C**)**. Firstly, supplement with Bcl-2 down-regulated the protein levels of cleaved-capase-2 and cleaved-capase-3 that had been up-regulated in PS-NPs-exposed Swan 71 cells and up-regulated Capase-3 protein levels that had been down-regulated in PS-NPs-exposed Swan 71 cells **(**Fig. 6D-G**)**. Meanwhile, supplement with Bcl-2 rescued (i.e. increased) the MMP levels in PS-NPs-exposed Swan 71 cells **(**Fig. 6H, I**)**. Moreover, supplement with Bcl-2 also alleviated (i.e. decreased) the apoptosis rate in PS-NPs-exposed Swan 71 cells **(**Fig. 6J, K**)**. Therefore, Bcl-2 played crucial roles in PS-NPs-induced trophoblast cell apoptosis; and supplement with Bcl-2 could effectively alleviate PS-NPs-induced trophoblast cell apoptosis.

### The levels of apoptosis were higher in RM vs. HC villous tissues

**Fig. 7:**
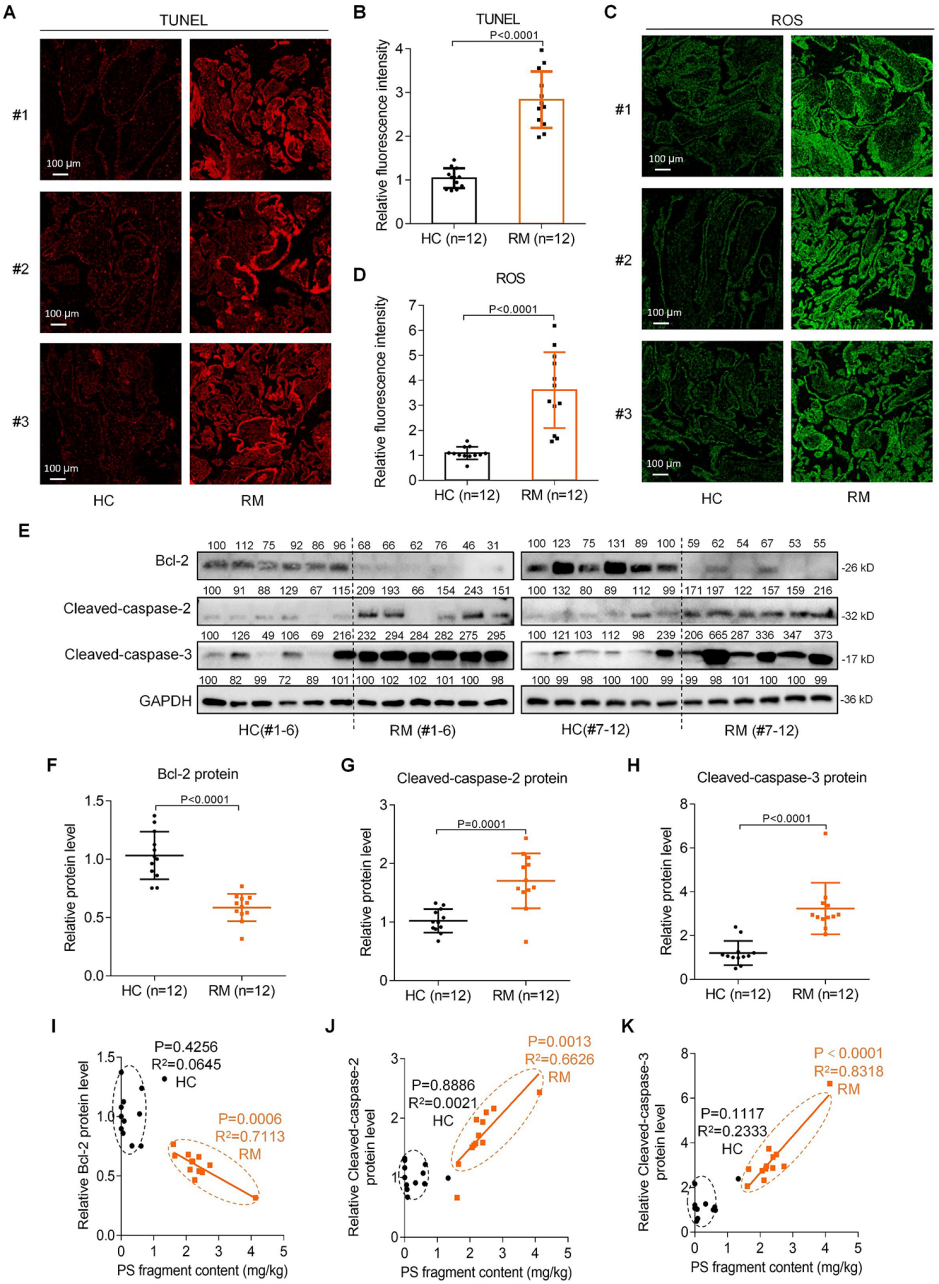
Higher levels of apoptosis and ROS in RM vs. HC villous tissues. (**A**-**B**) Representative images of TUNEL analysis of apoptosis levels in HC and RM villous tissues (scale bar, 100 μm) and their relative quantification of the fluorescence intensity (each *n* = 12). (**C**-**D**) Representative images of ROS assays in HC and RM villous tissues (scale bar, 100 μm) and their relative quantification of fluorescence intensity (each *n* = 12). (**E**-**H**) The protein levels of Bcl-2, Cleaved-caspase-2, and Cleaved-caspase-3 in HC and RM villous tissues (each *n* = 12). (**I**-**K**) The correlation analysis between the protein levels of Bcl-2 (**I**), Cleaved-caspase-2 (**J**), or Cleaved-caspase-3 (**K**) and PS fragment content in HC (round) and RM (square) groups (each *n* = 12)

Having known the presence of more PS plastic fragments in RM vs. HC villous tissues and PS-NPs-induced trophoblast cell apoptosis, next, we try to correlate PS plastic fragment exposure, apoptosis of villous tissues (including trophoblast cells), and miscarriage together. To this end, we analyzed the apoptosis levels of RM and HC villous tissues. TUNEL assays showed that the levels of apoptosis were significantly higher in RM vs. HC villous tissues (Fig. 7A). The levels of ROS were also higher in RM vs. HC villous tissues (Fig. 7B). These results confirmed that higher levels of apoptosis or ROS in villous tissues were positively associated with miscarriage. The protein levels of Bcl-2 were lower and those of Cleaved-caspase-2 and Cleaved-caspase-3 were higher in RM vs. HC villous tissues (Fig. 7C-F), which were consistent in the results obtained from PS-NPs-exposed trophoblast cells. Correlation analysis further showed that the protein levels of Bcl-2 were negatively correlated, whereas those of Cleaved-caspase-2 and Cleaved-caspase-3 were positively correlated, with the contents of PS plastic fragments in RM villous tissues (Fig. 7G-I). Collectively, these results indicated that PS plastic fragments exposure might be a risk factor for miscarriage, at least by inducing trophoblast cell apoptosis through Bcl-2/Caspase-2/Caspase-3 signaling pathway.

### PS-NPs exposure induced mouse miscarriage by increasing placental apoptosis

**Fig. 8:**
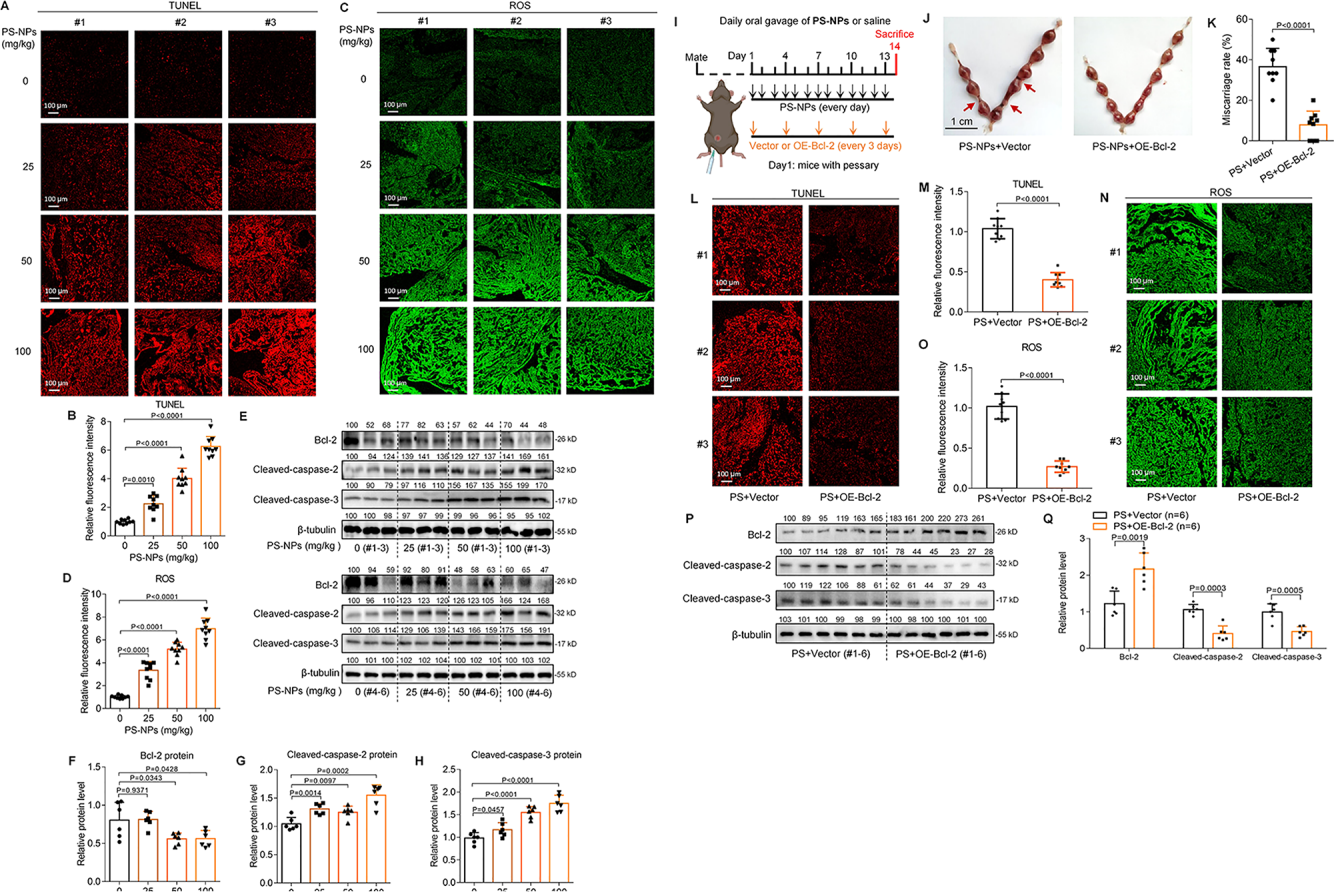
PS-NPs exposure increased placental apoptosis and thus induced mouse miscarriage. (**A**-**B**) Representative images of TUNEL analysis of apoptosis levels in PS-NPs-exposed mouse placental tissues (scale bar, 100 μm) and their relative quantification of fluorescence intensity (each *n* = 9). (**C**-**D**) Representative images of ROS assays in PS-NPs-exposed mouse placental tissues (scale bar, 100 μm) and their relative quantification of fluorescence intensity (each *n* = 9). (**E**-**H**) The protein levels of murine Bcl-2, Cleaved-caspase-2, and Cleaved-caspase-3 in placental tissues of PS-NPs-exposed mice (each *n* = 6). (**I**) Schematic diagram of mouse intervention assays. (**J**-**K**) The embryo adsorption and miscarriage rates in pregnant mice treated with PS-NPs + Vector or PS-NPs + OE-Bcl-2 (each *n* = 9, scale bar, 1 cm). (**L**-**M**) Representative images of TUNEL analysis of apoptosis levels in placental tissues of PS-NPs-exposed mice treated with PS-NPs + Vector or PS-NPs + OE-Bcl-2 (scale bar, 100 μm) and their relative quantification of fluorescence intensity (each *n* = 9). (**N**-**O**) Representative images of ROS assays in placental tissues of PS-NPs-exposed mice treated with PS-NPs + Vector or PS-NPs + OE-Bcl-2 (scale bar, 100 μm) and their relative quantification of fluorescence intensity (each *n* = 9). (**P**, **Q**) The protein levels of Bcl-2, Cleaved-caspase-2, and Cleaved-caspase-3 in placental tissues of mice treated with PS-NPs + Vector or PS-NPs + OE-Bcl-2 (each *n* = 6)

In PS-NPs-exposed mouse placental tissues, TUNEL assays showed that the levels of apoptosis in placental tissues were increased with increasing PS-NPs exposure concentrations **(**Fig. 8A**)**. Meanwhile, the levels of ROS in placental tissues were also increased with PS-NPs concentrations **(**Fig. 8B**)**. Sequence alignment analysis showed that Bcl-2, Caspase-2, and Caspase-3 genes are all evolutionarily conservative in human, mouse, rhesus, dogs, and elephants (Table S5). The protein levels of murine Bcl-2 were reduced, whereas those of Cleaved-caspase-2 and Cleaved-caspase-3 were increased, with increasing PS-NPs concentrations (Fig. 8C-F), indicating that PS-NPs exposure increased placental apoptosis and induced miscarriage by activating Bcl-2/Caspase-2/Caspase-3 signaling.

To explore the causality between apoptosis and miscarriage, we constructed a mouse miscarriage intervention model in which PS-NPs-exposed pregnant mice were intraperitoneally injected with murine Blc-2 overexpression plasmid, with vector plasmid as control, once per three days (Fig. 8G). Supplement with Blc-2 could efficiently reduce embryo adsorption and alleviated miscarriage rates (Fig. 8H, I). Meanwhile, supplement with Blc-2 also reduced the levels of apoptosis and ROS in placental tissues, as indicated by TUNEL and ROS assays (Fig. 8J, K). As for proteins, supplement with Blc-2 increased the protein levels of Bcl-2 and down-regulated the protein levels of Cleaved-caspase-2 and Cleaved-caspase-3 (Fig. 8L, M). These results supported that placental apoptosis induced miscarriage *in vivo.* Collectively, these results showed that PS-NPs exposure increased placental apoptosis, which further induced miscarriage, agreed with the results obtained from RM villous tissues and PS-NPs-exposed trophoblast cells.

## Discussion

### *Nanoplastics exposure induces miscarriage*

Increasing studies have demonstrated that PS-NPs exposure exhibits reproductive and developmental toxicity. PS-NPs can penetrate the chorion of zebrafish, accumulate in yolk sac, and further migrate to the gastrointestinal tract, gallbladder, liver, pancreas, heart, and brain throughout the whole development period of the offerings [75]. PS-NPs can also cross mouse placenta, result in defective neural tube morphogenesis, cause fetal lose, and increase mouse embryo resorption rates [32, 76–78], showing reproductive toxicity with different observation endpoints. For example, PS-NP exposure (oropharyngeal aspiration, 62.5 mg/kg, 80 nm in diameter, 3 times per week) during gestation for 21 days causes sex-specific small intestinal toxicity in offspring, which might contribute to reactive oxygen species activation and subsequent ferroptosis [77]. Exposure of Sprague Dawley rats (*n* = 5) to carboxylated polystyrene spheres (CPS-NPs, oral gavage, 2.5 mg/kg, 25 nm in diameter) on gestational day 19 for 24 h shows that CPS-NPs can breach the intestinal barrier and maternal-fetal barrier of the placenta to access the fetal circulation and all fetal tissues [78]. Exposure of mice to PS-NPs (intravenous injection, 15 mg/kg, 60 nm in diameter) on gestational days 9.5 and 15 shows that PS-NPs can cross mouse placentas and affect the development of mouse fetuses [32]. In this study, using mouse model, we find that exposure to 50 nm PS-NPs (such as 50 or 100 mg/kg/d) during pregnancy for continuous 14 days indeed induces mouse miscarriage. However, exposure to 25 mg/kg PS-NPs does not show obvious miscarriage. Taken together, we propose that different exposure routes, doses, and periods, and particle sizes of PS-NPs might give different observed endpoints. Recently, PS-MPs with different sizes have been detected out from human placenta, blood, and feces [16, 17]. Villous tissues are the main tissues for nutrient exchange in placenta. In this study, PS plastics particles were detected out from villous tissues, and their contents are higher in unexplained RM vs. HC villous tissues, and the higher contents of PS plastics particles in villous tissues are positively associated with miscarriage. Therefore, we conclude that exposure to excessive PS-NPs is a non-negligible risk factor for women unexplained miscarriage.

*Nanoplastics exposure causes placental apoptosis and induces miscarriage.* Trophoblasts, the key cells at the maternal-fetal interface, play an essential role in placenta implantation and reproduction [79, 80]. Excessive apoptosis of trophoblast cells is associated with various adverse pregnancy outcomes, such as preeclampsia [81], miscarriage, and fetal growth restriction [82]. What’s more, trophoblast cells are very sensitive to environmental toxicants. It has been discovered that exposure to triazole fungicide tebuconazole, bisphenol A, heavy metal cadmium, or perfluorooctanoic acid can induce trophoblast cell apoptosis [44–47]. As for PS-NPs, transcriptome sequencing analysis shows that PS-NPs exposure is associated with human trophoblast cell apoptosis [55] and perfused human placenta apoptosis [56]. PS-NPs, including PS-NPs with surface modification (such as -NH_2_ or -COOH), induce trophoblast cell apoptosis by up-regulating cleaved-caspase 3 or down-regulating Bcl-2 [57, 58]. Another study also reports that co-exposure to PS-MPs and PS-NPs injures fetal thalamus by inducing cell apoptosis by up-regulating cleaved-caspase-3 [52]. In this study, we further confirm that PS-NPs exposure causes apoptosis and then induces miscarriage by activating Bcl-2/Cleaved-caspase-2/Cleaved-caspase-3 signaling through mitochondrial pathway. Supplement with Bcl-2 can efficiently reduce apoptosis and alleviate miscarriage in PS-NPs-exposed pregnant mouse model. All these studies demonstrate that PS-NPs cause placental apoptosis and induce miscarriage.

**Fig. 9:**
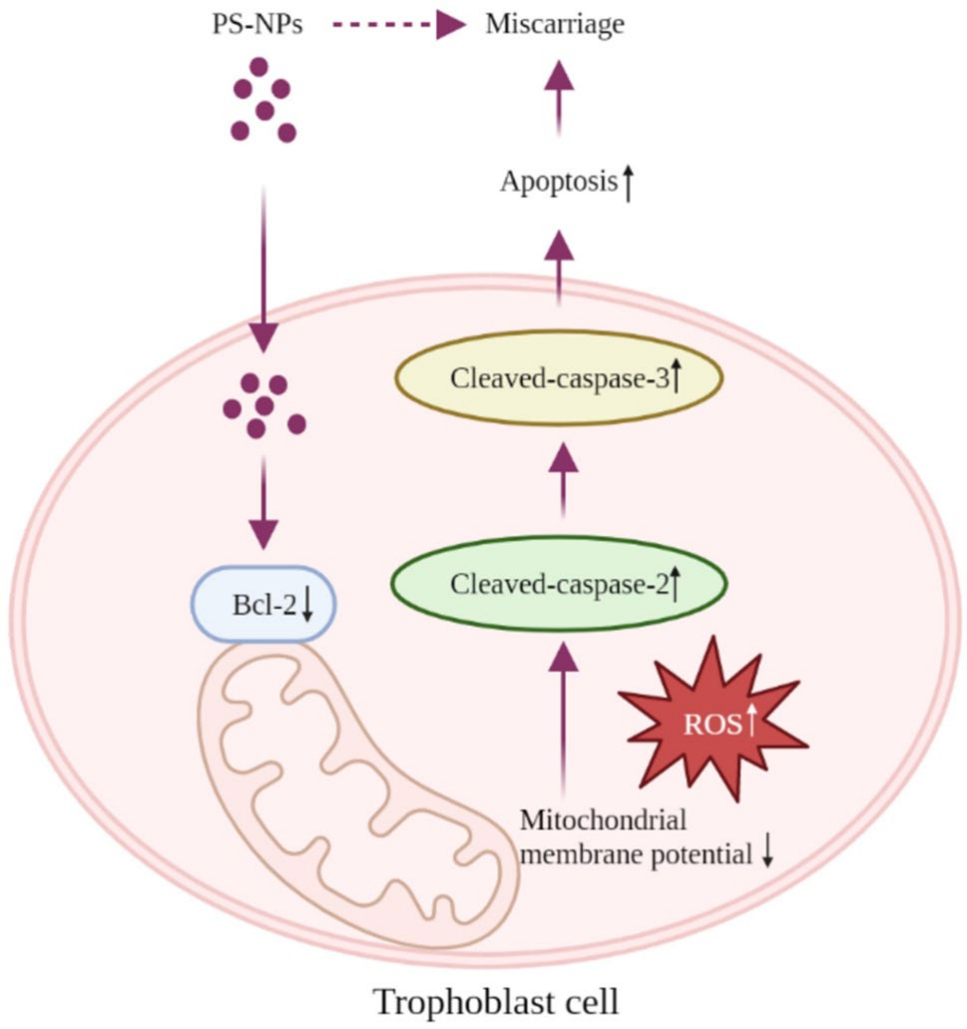
The proposed regulatory mechanism. PS-NPs can readily enter trophoblast cells and distribute primarily in cytoplasm, where PS-NPs activate Bcl-2/Cleaved-caspase-2/Cleaved-caspase-3 signaling through the classical mitochondrial pathway, leading to trophoblast cell apoptosis and further inducing miscarriage

The potential mechanism underlying PS-NPs-induced apoptosis is proposed (Fig. 9). PS-NPs can readily enter trophoblast cells, distribute primarily in cytoplasm, and promote trophoblast cell apoptosis at a dose- and time-dependent manner. Firstly, exposure to PS-NPs reduces the expression levels of Bcl-2, disrupts mitochondrial membrane permeability, resulting in a decrease in MMP and an increase in intracellular ROS, and ultimately activates Caspase-2 and Caspase-3. Therefore, exposure to PS-NPs activates Bcl-2/Cleaved-caspase-2/Cleaved-caspase-3 signaling pathway through the classical mitochondrial pathway, leading to trophoblast cell apoptosis. As a key molecule, supplement with Bcl-2 could effectively protect Swan 71 cells from PS-NPs-induced apoptosis. Moreover, the down-regulation of Bcl-2 and up-regulation of Cleaved-caspase-2 and Cleaved-caspase-3 are also observed in villous tissues of unexplained RM patients, also in the placental tissues of PS-NPs-exposed pregnant mice with miscarriage. Supplement with murine Bcl-2 can efficiently reduce placental apoptosis and alleviate miscarriage in PS-NPs-exposed mice. Therefore, assays using cells, tissues, and mouse model show that PS-NPs exposure induces apoptosis in placenta tissues (including trophoblast cells) by activating Bcl-2/Cleaved-caspase-2/Cleaved-caspase-3 pathway and then induces miscarriage.

### *PS particles might cause women reproductive toxicity*

A report has shown that the median intake of MPs (1-5000 μm) is estimated as 4.1 µg/week (or 0.58 µg/day) for an adult by correcting their actual contents in foods [20]. NPs concentrations are estimated to be about 2.4 × 10^5^ particles per liter of bottled water [83], which indicates that an adult intakes approximately 5 × 10^5^ NPs daily through drinking bottled water. However, NPs with smaller sizes was relatively harder to be detected in tissues and their total amount in human bodies was still largely unclear. Moreover, MPs and NPs are difficult to metabolize in human body and may be accumulated [8, 84]. In mouse assays, exposure to 25 mg/kg/day 50 nm PS-NP for 14 days did not show the obvious miscarriage but indeed induced placental apoptosis, and exposure to 50 and 100 mg/kg/day showed obvious placental apoptosis and miscarriage. Notably, the doses of PS-NPs used for mouse assays were really much higher than the actual detected dose in human, indicating that exposure to PS-NPs at higher dose could cause trophoblast cell apoptosis and induce miscarriage. However, considering the unclear amount of PS-NPs with smaller sizes, which generally have more toxic effects than MPs with larger sizes [85], the long-term exposure of plastic particles produces higher levels of apoptosis and ROS, which might produce productive toxicity and cause other adverse pregnancy outcomes. Moreover, analysis of the contents of PS particles in 18 pair of HC and UM villous tissues has showed that the contents in villous tissues were positively associated with miscarriage and could be considered as a risk factor for miscarriage. However, the actual exposure dose that directly induce women miscarriage should be further explored.

### *Prospects*

In this study, we mainly focused on the effects of PS-NP exposure during pregnancy on miscarriage. The effects of PS-NP exposure during pre-pregnancy and pregnancy on other adverse pregnancy outcomes or offspring should also be investigated to discover more comprehensive reproductive toxicity of PS-NPs. In this study, we used regular and spherical 50 nm PS-NPs as a model of PS particles. In reality, plastic particles were made of various materials (such as polyethylene, polypropylene, or polyethylene terephthalate) with different sizes, shapes, and surface modifications. These real characteristics should be considered for their reproductive toxicity. In fact, other types of plastic fragments were also detected out in women villous tissues by Py-GC/MS, such as polyethylene and polyvinyl chloride. However, their abundance is lower than that of PS in these samples. The toxic effects of other types of plastic particles should also be considered in the further. More importantly, it was unknown whether PS-NPs might participate in chemical reactions within cells to generate new surface modification. Whether these newly modified PS-NPs might participate in the regulation of cell functions still needs further exploration.

### *Effective preventive approaches*

Microplastics pollution has become global environmental concerns. Human, including pregnant women as vulnerable and susceptible populations, should reduce the use of disposable plastics, classify and recycle toxic and ordinary plastics, and do not dispose of any plastic products casually. In addition, there is an urgent need to develop new degradation strategies to achieve efficient removal of MPs and NPs, mainly including biodegradation, advanced oxidation degradation, and photocatalytic degradation. Alternatively, the alternatives to plastics are also required to be developed.

## Conclusion

In this study, we identify that polystyrene plastics particles are present in women villous tissues and their higher contents in villous tissues are associated with miscarriage. We also discover that exposure to PS-NPs with doses of 50 or 100 µg/kg indeed induce miscarriage by increasing placental apoptosis in a PS-NPs-exposed pregnant mouse model. In mechanism, PS-NPs exposure increases oxidative stress, decreases mitochondrial membrane potential, and increases apoptosis in human trophoblast cells by activating Bcl-2/Cleaved-caspase-2/Cleaved-caspase-3 signaling through mitochondrial pathway. Supplement with Bcl-2 can efficiently suppress apoptosis in PS-NPs-exposed trophoblast cells and suppress placental apoptosis and alleviate miscarriage in PS-NPs-exposed mouse model. This study reveals the presence of polystyrene plastics particles in women villous tissues, their new toxicological effects, and the novel underlying mechanism, indicating that plastics particle exposure is a new risk factor for women unexplained miscarriage.

### Electronic supplementary material

Below is the link to the electronic supplementary material.

Supplementary Material 1

Supplementary Material 2

## Data Availability

All data and materials are included in the manuscript.

## Abbreviations

MPs: microplastics
PS-NPs: polystyrene nanoplastics
HC: healthy control
RM: recurrent miscarriage
CI: confidence interval
OR: odds ratio
ROC: Receiver Operating Characteristic
SD: standard deviation
AUC: Area Under Curve
FITC: fluorescein isothiocyanate
ROS: reactive oxygen species
MMP: Mitochondrial membrane potential
TEM: transmission electron microscope
Py-GC/MS: pyrolysis-gas chromatography/mass spectrometry
Fer-1: ferrostatin-1
Nec-1: Necrosstatin-1
