# Supplementary figures and images for: Exposure to high dose of polystyrene nanoplastics causes trophoblast cell apoptosis and induces miscarriage
Source: Part Fibre Toxicol. 2024 Mar 7;21:13. doi: 10.1186/s12989-024-00574-w (PMC10921758; doi:10.1186/s12989-024-00574-w)

## Slide 1
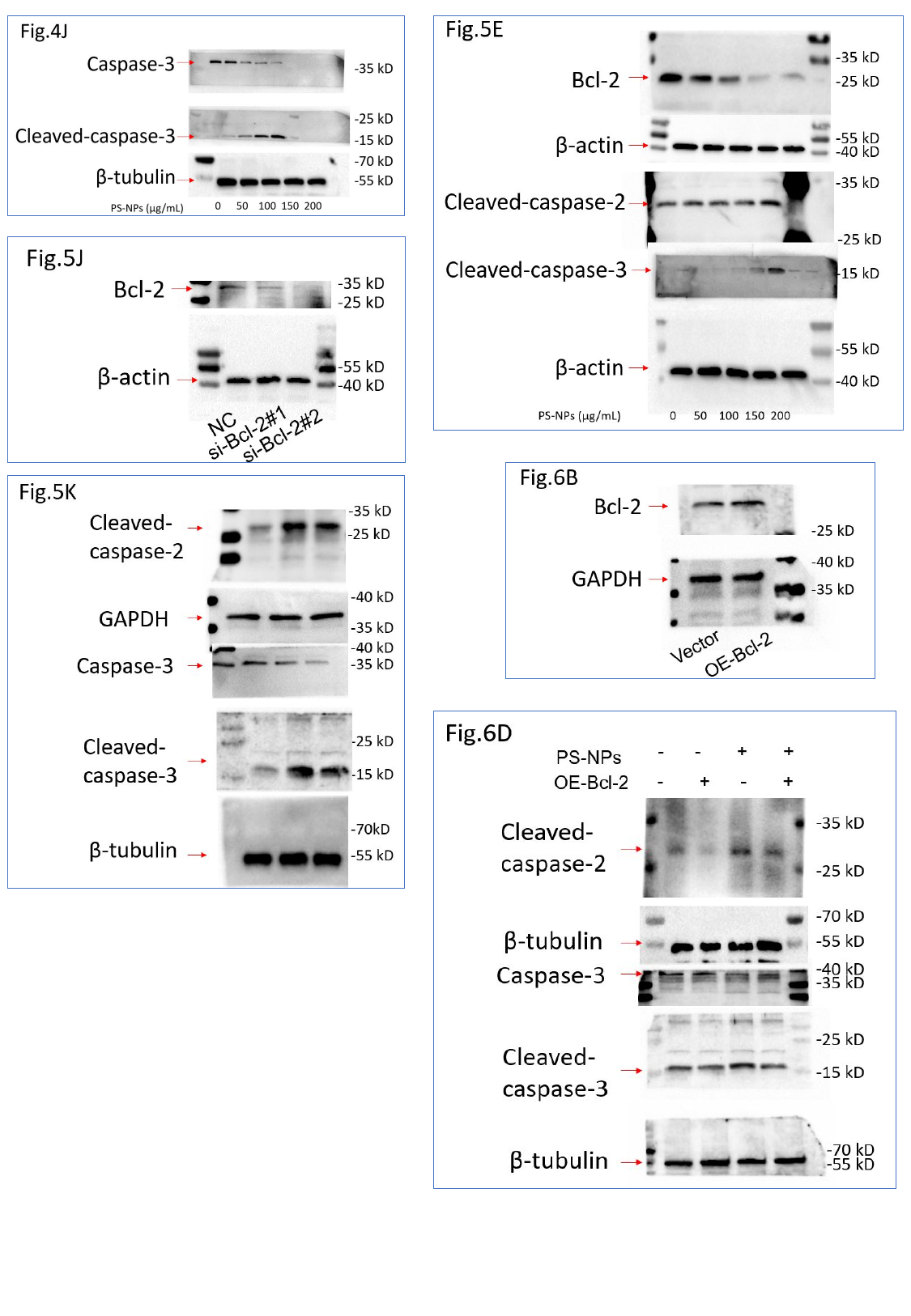

## Slide 2
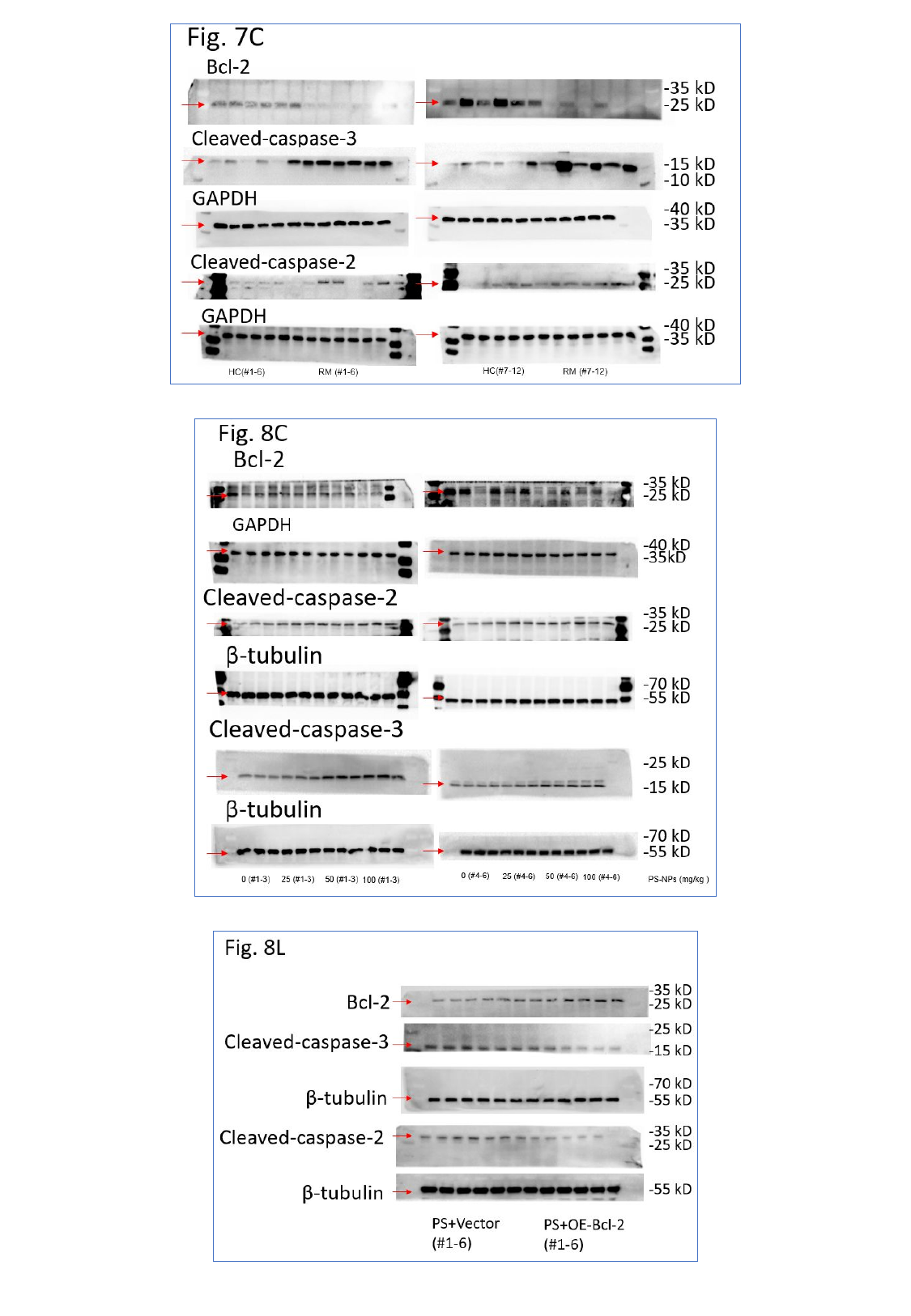

Supplement: Supplementary file 1 — Supplementary Material 1 [file 12989_2024_574_MOESM1_ESM.pptx]
