# Supplementary material for: Exposure to high dose of polystyrene nanoplastics causes trophoblast cell apoptosis and induces miscarriage
Source: Part Fibre Toxicol. 2024 Mar 7;21:13. doi: 10.1186/s12989-024-00574-w (PMC10921758; doi:10.1186/s12989-024-00574-w)
Supplement: Supplementary file 2 — Supplementary Material 2 [file 12989_2024_574_MOESM2_ESM.docx]

Supporting information

**Exposure to high dose of polystyrene nanoplastics causes trophoblast cell apoptosis and induces miscarriage**

Shukun Wan^1,2#^, Xiaoqing Wang^1,2#^, Weina Chen^1,2#^, Manli Wang^1^, Jingsong Zhao^1^, Zhongyan Xu^1^, Rong Wang^1^, Chenyang Mi^1^, Zhaodian Zheng^1^, and Huidong Zhang^1^*

Table of Contents

**Table S1**. Primers used for RT-qPCR in this study.

**Table S2**. RNA sequences used for RNA transfection.

**Table S3**. DNA sequences used for construction of pcDNA3.1 overexpression plasmids.

**Table S4** Characteristics of RM and HC women in this study (n=18).

**Table S5**. Sequence conservation analysis of human mRNAs and proteins with those in other species.

**Table S1.** Primers used for RT-qPCR in this study.

|  | Gene | Forward (5'-3') | Reverse (5'-3') | |
| --- | --- | --- | --- | --- |
| Human | Bcl-2 | GGTGGGGTCATGTGTGTGG | | CGGTTCAGGTACTCAGTCATCC |
|  | GAPDH | GTCTCCTCTGACTTCAACAGCG | | ACCACCCTGTTGCTGTAGCCAA |
| Mouse | Bcl-2 | GCTACCGTCGTGACTTCGC | | CCCCACCGAACTCAAAGAAGG |
|  | Gapdh | TGGCCTTCCGTGTTCCTAC | | GAGTTGCTGTTGAAGTCGCA |

**Table S2**. RNA sequences used for RNA transfection.

| Gene | Sense (5'-3') | Antisense (5'-3') |
| --- | --- | --- |
| si-Bcl-2#1 | GGGAGAUAGΜGAΜGAAGUATT | UACUUCAUCACUAUCUCCCTT |
| si-Bcl-2#2 | GGAGAACAGGGUACGAUAATT | UUAUCGUACCCΜGUUCUCCTT |
| NC | UUCUCCGAACGUGUCACGUTT | ACGUGACACGUUCGGAGAATT |

**Table S3**. DNA sequences used for construction of pcDNA3.1 overexpression plasmids.

|  | Plasmid name | Gene name | Sequence region |
| --- | --- | --- | --- |
| Human | pcDNA3.1-Bcl-2 | Bcl-2 | CDS region ([NM_000633.3](https://www.ncbi.nlm.nih.gov/nuccore/NM_000633.3)) |
| Mouse | pcDNA3.1-Bcl-2 | Bcl-2 | CDS region (NM_009741.5) |

**Table S4 Characteristics of RM and HC women in this study (n=18).**

| Characteristics | RM | HC | P value |
| --- | --- | --- | --- |
| Maternal Age (years) | 27.0 ± 4.03 | 27.8 ± 3.80 | 0.568 |
| Pre-pregrancy BMI (kg/m^2^) | 22.7 ± 4.67 | 21.8 ± 3.05 | 0.440 |
| Gestational days | 51.5 ± 6.45 | 52.5 ± 5.93 | 0.843 |
| Gestational weeks | 7.02 ± 1.26 | 7.52 ± 1.06 | 0.406 |
| RBC (10^12^/L) | 4.34 ± 0.46 | 4.26 ± 0.67 | 0.845 |
| WBC (10^9^/L) | 6.65 ± 1.25 | 6.23 ± 1.45 | 0.428 |
| Hemoglobin (g/L) | 133 ± 5.95 | 132 ± 6.92 | 0.420 |

Abbreviations: RM: Recurrent miscarriage group (n=18); HC: Healthy control group (n=18); P value: Chi-square test; RBC: Red blood cell; WBC: White blood cell.

**Table S5**. Sequence conservation analysis of human mRNAs and proteins with those in other species^a^

|  |  |  | Mouse | Rhesus | Dog | Elephant |
| --- | --- | --- | --- | --- | --- | --- |
| mRNA | Bcl-2 | Per.Ident^b^ | 87.9% | 96.5% | 89.9% | 89.7% |
|  | Caspase-2 | Per.Ident^b^ | 89.0% | 96.3% | 89.0% | 87.7% |
|  | Caspase-3 | Per.Ident^b^ | 85.5% | 94.8% | 88.8% | 87.2% |
| Protein | Bcl-2 | Per.Ident^b^ | 93.0% | 98.8% | 94.1% | 95.6% |
|  | Caspase-2 | Per.Ident^b^ | 93.1% | 98.1% | 92.2% | 88.0% |
|  | Caspase-3 | Per.Ident^b^ | 88.6% | 96.4% | 90.6% | 88.1% |

^a^Sequence conservation of Bcl-2, Caspase-2, and Caspase-3 was explored using NCBI Blast (https://blast.ncbi.nlm.nih.gov/Blast.cgi).

^b^Per.Ident means the ratio of the number of the matched ribonucleotides or amino acid residues to the total ribonucleotides or amino acid residues in the sequence.


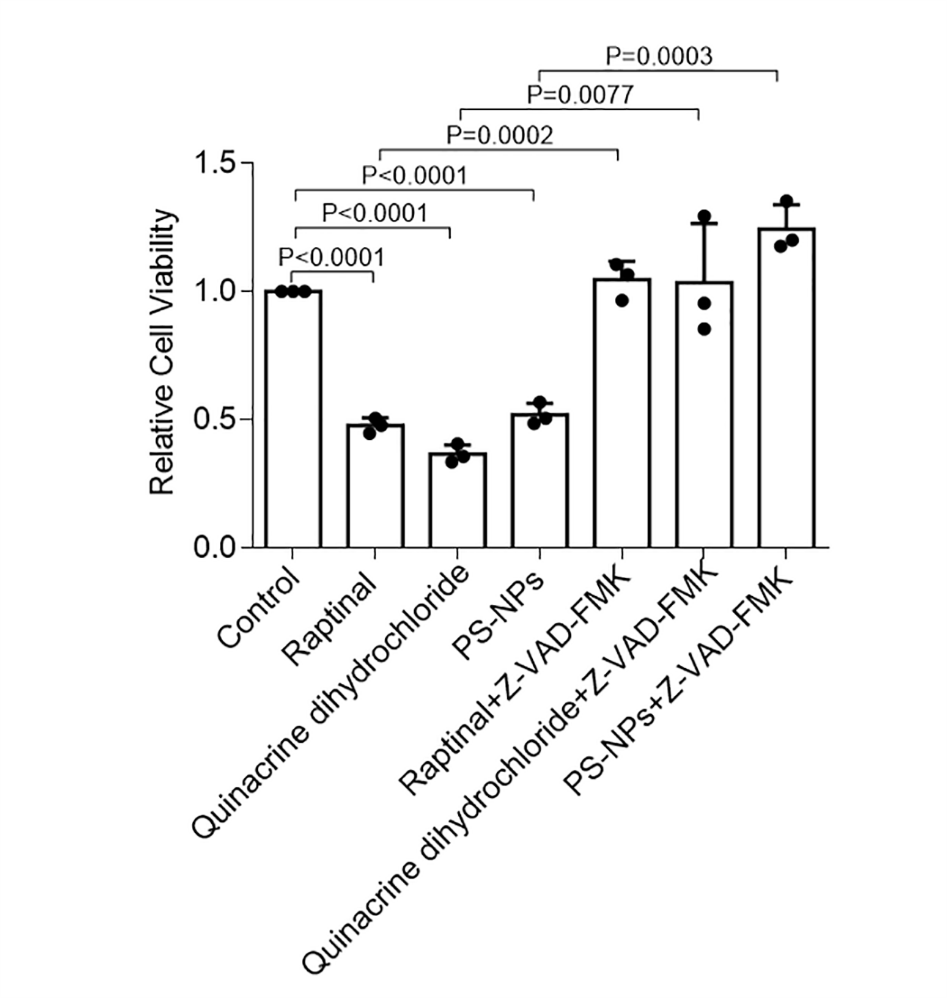


**Fig. S1.** Cell viability of trophoblast cells treated with 10 μM Raptinal, 15 μM Quinacrine dihydrochloride, or 500 μg/mL PS-NPs, and co-treated with 10 μM Z-VAD-FMK for 48 h.
